# MARCO expression on myeloid-derived suppressor cells is essential for their differentiation and immunosuppression

**DOI:** 10.1038/s41420-025-02627-1

**Published:** 2025-07-22

**Authors:** Sijia Liu, Binle Tian, Na Wang, Zhilong Wang, Wen Zhang, Qi Li, JianFei Wang, Guo-Huang Fan, Caicun Zhou

**Affiliations:** 1 Department of Medical Oncology, Shanghai Pulmonary Hospital, Tongji University School of Medicine, Shanghai, China; 2 Cancer Center, Shanghai General Hospital, Shanghai Jiao Tong University School of Medicine, Shanghai, China; 3 Shanghai Key Laboratory of Pancreatic Disease, Institute of Pancreatic Disease, Shanghai Jiao Tong University School of Medicine, Shanghai, China; 4 Department of Antibody Development, Immunophage Biotech Co., Ltd, Shanghai, China; 5 Department of Oncology, Immunophage Biotech Co., Ltd, Shanghai, China; 6 Executive Office, Immunophage Biotech Co., Ltd, Shanghai, China; 7 Shanghai Laboratory Animal Research Center, Shanghai, China; 8 Department of Oncology, Shanghai East Hospital, Tongji University School of Medicine, Shanghai, China

**Keywords:** Cancer microenvironment, Breast cancer, Cancer immunotherapy

## Abstract

Myeloid-derived suppressor cells (MDSCs) significantly contribute to the immunosuppressive tumor microenvironment (TME), and targeted inhibition of MDSCs is a potential therapeutic strategy against cancer. Here, we identify macrophage receptor with collagenous structure (MARCO) as a critical regulator of MDSC differentiation and immunosuppression in breast cancer. The present study demonstrates that MARCO is expressed on MDSCs, and breast tumor-derived exosomes (TDEs) enriched with macrophage migration inhibitory factor (MIF) promote MDSC differentiation and amplify immunosuppressive activity by up-regulating MARCO. Genetic ablation of MARCO in a murine breast cancer model attenuated tumor growth, accompanied by reduced monocytic MDSCs (M-MDSCs) and total tumor-associated macrophages (TAMs), along with enhanced infiltration of CD8^+^ T cells and natural killer (NK) cells. Furthermore, we developed a specific MARCO down-regulation-promoting monoclonal antibody that impeded TDE-induced MDSC differentiation and immunosuppression. In vivo, MARCO down-regulating antibody suppressed tumor growth and reprogrammed the TME by diminishing immunosuppressive MDSCs and TAMs and revitalizing CD8^+^ T cells and NK cells. Strikingly, combining the MARCO down-regulating antibody with PD-1 blockade synergistically enhanced anti-tumor efficacy. This work establishes MARCO as a key regulator of MDSC-mediated immunosuppression and presents a compelling case for the inclusion of MARCO as a therapeutic target in cancer immunotherapy.

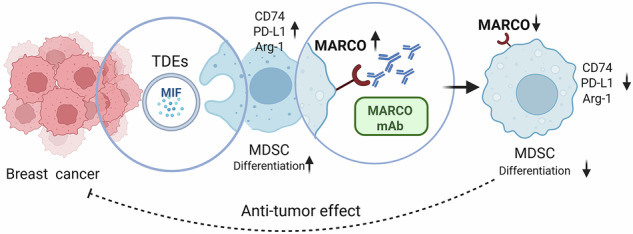

## Introduction

The tumor microenvironment (TME) represents a complex and dynamic system comprising diverse cellular and noncellular components [1]. Within this intricate network, immune cells play a pivotal role in tumor progression and immune evasion. These immune populations include both adaptive immune cells, such as T cells and B cells, as well as innate immune cells, including macrophages, myeloid-derived suppressor cells (MDSCs), and natural killer (NK) cells at the interface between innate and adaptive immunity [2, 3]. The TME induces functional alterations in immune cells, reprogramming them to either diminish inflammatory responses or enhance immunosuppression. One of the most prevalent mechanisms by which cancer subverts immune responses is through immunosuppression by MDSCs [4]. MDSCs are a population of immature myeloid cells that acquire potent immunosuppressive properties as tumor development [5–7]. These cells are typically classified into two subsets based on their myeloid origin: granulocytic/polymorphonuclear MDSCs (G-MDSC/PMN-MDSCs) and monocytic MDSCs (M-MDSCs) [8]. Studies have revealed that while G-MDSCs account for the majority of total MDSCs, the less abundant M-MDSCs demonstrate significantly stronger immunosuppressive activity [5, 9–11]. MDSCs exert suppressive effects via multiple mechanisms, including upregulation of Nos2 and arginase 1 (Arg-1) to inhibit CD8^+^ T cell activation, and secretion of immunosuppressive cytokines such as IL10 to promote regulatory T cells (Tregs) and induce macrophages polarized toward M2-like phenotype [10, 12, 13].

The macrophage receptor with collagenous structure (MARCO), a member of the class A scavenger receptor family [14–16], has recently been revealed as an important immunomodulator in tumor-associated macrophages (TAMs), particularly within the M2-like sub-population. Clinical studies across multiple cancer types, including pancreatic ductal adenocarcinoma (PDAC), colon cancer, and breast cancer, have consistently demonstrated that increased infiltration of MARCO^+^ TAMs correlates with poor patient prognosis [17–21]. While the immunosuppressive functions of MARCO^+^ TAMs have been partially elucidated, such as their role in enhancing the proliferation of Tregs while diminishing CD8^+^ T cells and NK cells [21, 22], the expression and biological significance of MARCO in other myeloid cells remain controversial. Initial reports suggested MARCO expression was restricted to TAMs and absent in lymphocytes or other myeloid subsets [21]. However, there is evidence that indicates that tumor conditions MARCO^+^ macrophages exhibit MDSC-like features [23], raising the intriguing possibility that MARCO may contribute to the immunosuppressive phenotype of MDSCs. This potential connection between MARCO expression and MDSC warrants further investigation to elucidate its role in shaping the immunosuppressive tumor microenvironment.

To validate this hypothesis, we first confirmed MARCO expression on MDSCs. We subsequently demonstrated that tumor-derived exosomes (TDEs) induce MDSC differentiation accompanied by elevated MARCO expression and enhanced immunosuppression. Mechanistically, we identify macrophage migration inhibitory factor (MIF) in TDEs as the key mediator of these effects, evidenced by both knocking down MIF expression in TDEs and pharmacological inhibition using the small molecule inhibitor IPG1576. In vivo studies using MARCO deficiency mice demonstrated significant tumor growth suppression that was associated with marked reduction of intratumoral M-MDSCs and M2-like TAMs, and enhanced accumulation of CD8^+^ T cells and NK cells. We then developed a novel MARCO monoclonal antibody that potently downregulated MARCO expression. This MARCO antibody inhibited TDE-induced MDSC differentiation and their immunosuppressive activity in vitro. More importantly, in a murine breast cancer model, the MARCO downregulating antibody exhibited significant anti-tumor activity and synergized with PD-1 blockade. These findings establish MARCO-targeted immunotherapy as a promising new approach for cancer treatment.

## Results

### TDE induces MARCO expression and promotes MDSC differentiation

**Fig. 1:**
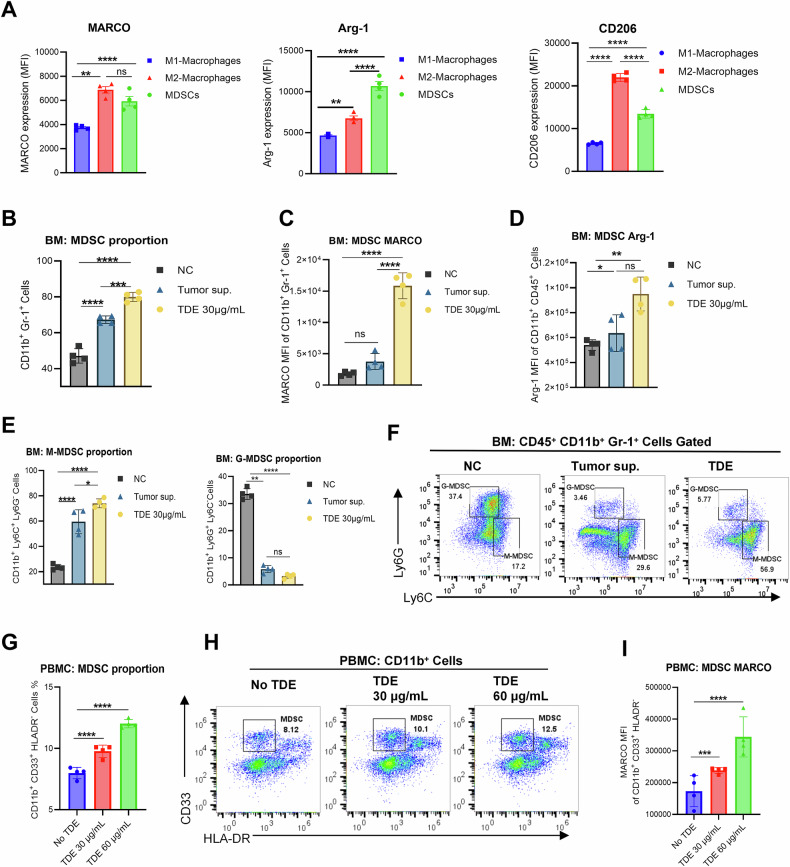
TDE induces MARCO expression and promotes MDSC differentiation. **A** Flow cytometric analysis of MARCO, Arg-1, and CD206 expression on cytokine-induced murine bone marrow cells. (M1: M-CSF + LPS + IFN-γ; M2: M-CSF + IL4 + IL10; MDSC: GM-CSF + IL6). **B**–**D** MDSC proportion, MDSC MARCO expression, and MDSC Arg-1 expression on murine bone marrow cells treated with E0771 tumor-sup or TDEs. **E** M-MDSC and G-MDSC proportion. Murine BM cells treated with E0771 tumor-sup or TDE. **F** Representative flow cytometry figure of MDSC subgroups. **G** Human PBMC cells were treated with different concentrations of MDA-MMB-231 TDE. Then, the MDSC proportion was detected. **H** Representative flow cytometry figure of MDSC proportion. **I** MARCO expression on human TDE-induced MDSC. Data are presented as mean ± SD with a CI of 95% from three biological replicates (individual data points overlaid), and the entire study was independently repeated at least three times. *p* < 0.05 by one-way ANOVA with Tukey’s multiple comparisons test. *p < 0.05, **p < 0.01, ***p < 0.001, ****p < 0.001.

It has been reported that MARCO plays a role in M2 macrophage infiltration in the tumor tissue and affects the prognosis of breast cancer [21]. However, the relationship between MARCO and MDSC has not yet been adequately studied. We thus compared MARCO expression across polarized myeloid subsets. The result revealed that higher MARCO expression was observed on MDSCs and M2 macrophages (Fig. 1A). In addition, MARCO-expressing myeloid cells also exhibited immunosuppressive characteristics, with Arg-1 highly expressed on MDSCs and CD206 overexpressed on M2 macrophages. Multiplex immunofluorescence (m-IF) analysis of human triple-negative breast cancer (TNBC) tissues demonstrated co-localization of MARCO with CD11b^+^ CD14^+^ cells that were negative for CD68 expression, suggesting potential expression of MARCO on M-MDSCs (Fig. S1A, B). Additionally, patients with high MARCO expression (patients with total MARCO^+^ cell count per whole slide ≥ median value) exhibited significant enrichment of M-MDSCs (CD11b^+^ CD14^+^ CD15^−^) and G-MDSCs (CD11b^+^ CD15^+^ CD14^−^) (Fig. S1C–E).

Based on the findings of MARCO expression on MDSCs, we conducted an in vitro experiment to elucidate the relationship of MARCO expression with MDSC differentiation. We isolated TDEs from E0771 breast cancer cell supernatant and validated their identity by CD9, CD63, and GAPDH expression, nanoparticle tracking analysis (NTA), and transmission electron microscopy (TEM) (Fig. S2A–D). Murine bone marrow cells (BM) were then treated with either crude E0771 tumor supernatant (Tumor-sup.) or purified TDEs (30 μg/mL) in the presence of M-CSF (40 ng/mL) for 4 days. We found that both E0771 supernatant and TDEs significantly promoted MDSC differentiation (Fig. 1B). Strikingly, E0771 TDEs exhibited a stronger induction capacity. TDE-induced MDSCs exhibited markedly higher expression of MARCO and Arg-1 (Fig. 1C, D). Further, analysis of MDSC subgroups revealed a concurrent elevation of M-MDSCs (CD11b^+^ Ly6C^+^ Ly6G^−^) and decline of G-MDSCs (CD11b^+^ Ly6G^+^ Ly6C^−^) in tumor conditions. And the evaluation of M-MDSCs was more pronounced in the TDE group than in the tumor supernatant group (Fig. 1E, F), implicating that TDEs induce MARCO upregulation and M-MDSC differentiation.

To validate these findings, we exposed human peripheral blood mononuclear cells (PBMCs) to increasing concentrations of MDA-MB-231-derived TDEs (0, 30, 60 μg/mL). Consistent with the findings in murine, TDEs dose-dependently increased the proportion of total MDSCs (CD11b^+^ CD33^+^ HLA-DR^−^), with a pronounced upregulation of MARCO (Fig. 1G–I). These results collectively demonstrate that breast cancer TDEs universally promote MARCO-dependent MDSC differentiation.

### MIF in TDE promotes MDSC differentiation and MARCO upregulation

**Fig. 2:**
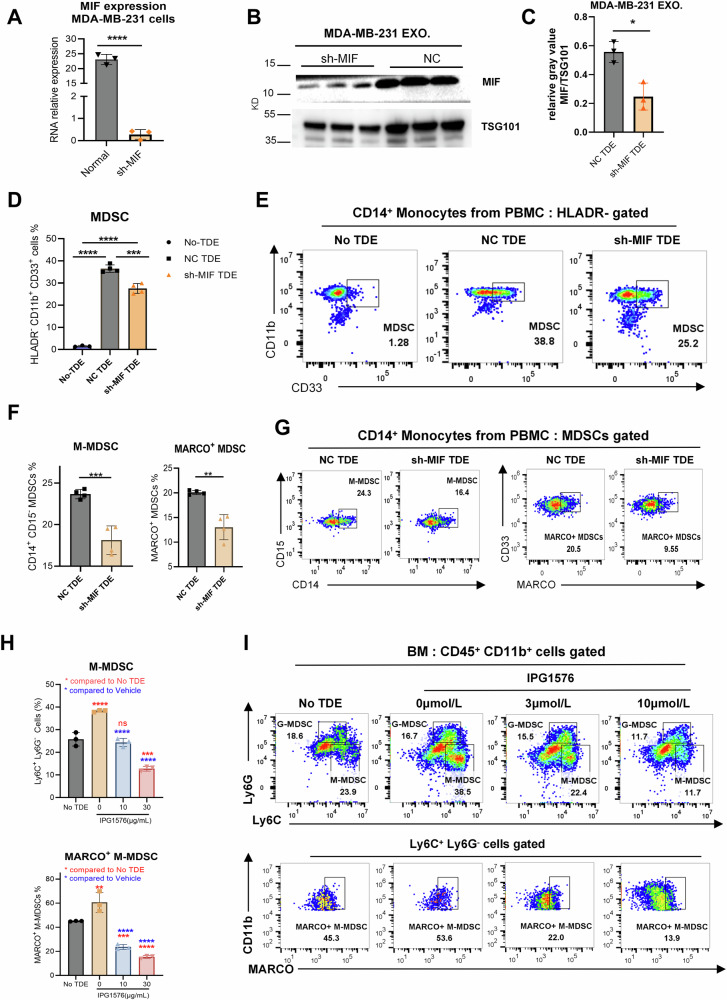
MIF in TDE promotes MDSC differentiation and MARCO upregulation. **A** MIF RNA expression of MDA-MBA-231 cells transfected with different indicated plasmids. **B** Representative Western blot images demonstrate the expression of MIF in TDEs isolated from MDA-MB-231 transfected with MIF shRNA (sh-MIF) or negative control shRNA (NC). Three biological replicates per group. **C** Relative gray value analysis of Western blot images to prove MIF knockdown in exosomes. **D** The proportions of MDSCs when human PBMCs respond to the sh-MIF or NC TDEs. Each experiment included four biological replicates, and the entire study was independently repeated at least three times. **E** Representative flow cytometry figure of (**D**). **F** The proportions of human PBMC differentiated M-MDSCs and MARCO^+^ MDSC in response to the sh-MIF or NC TDEs. **G** Representative flow cytometry figure of (**F**). **H** Mouse BM cells induced by E0771 exosomes were treated with IMP-1576 with different concentrations, compared to BM without E0771 TDE. Quantitation data of M-MDSC and MARCO^+^ MDSC proportion. **I** Representative flow cytometry figure of (**H**). Data was shown as mean ± SD. with a CI of 95% and individual data points overlaid. *p* < 0.05 by one-way ANOVA with Tukey’s multiple comparisons test for pairwise comparisons between groups. **p < 0.01, ***p < 0.001, ****p < 0.001. ns: no significance.

MIF is the main component of TDEs, inducing monocyte differentiation into MDSCs. Therefore, we will further elucidate whether MIF in TDEs is a critical component leading to MARCO upregulation and MDSC differentiation. To confirm the hypothesis, we performed MIF knockdown in MDA-MB-231 human breast cancer cells using shRNA technology (sh-MIF), with scrambled shRNA serving as a negative control (NC). RT-PCR and Western blot confirmed successful MIF depletion in sh-MIF-transfected cells (Fig. 2A). and isolated TDEs (Fig. 2B, C). We found that sh-MIF TDEs significantly attenuated MDSC differentiation, compared to NC TDEs (Fig. 2D, E). Further, MIF depletion in TDEs abolished M-MDSC expansion and MARCO upregulation (Fig. 2F, G).

To pharmacologically validate the role of MIF in TDEs, we used IPG1576, a highly potent MIF inhibitor targeting MIF in TDEs [24]. Notably, IPG1576 reversed the differentiation of MDSC and MARCO expression induced by E0771 TDE in a dose-dependent way (Fig. 2H, I). Collectively, these data suggest that MIF in TDEs is essential for the differentiation of monocytes into immunosuppressive MDSCs and triggers MARCO upregulation.

### MARCO deficiency prevented tumor growth and altered the characteristics of MDSCs

**Fig. 3:**
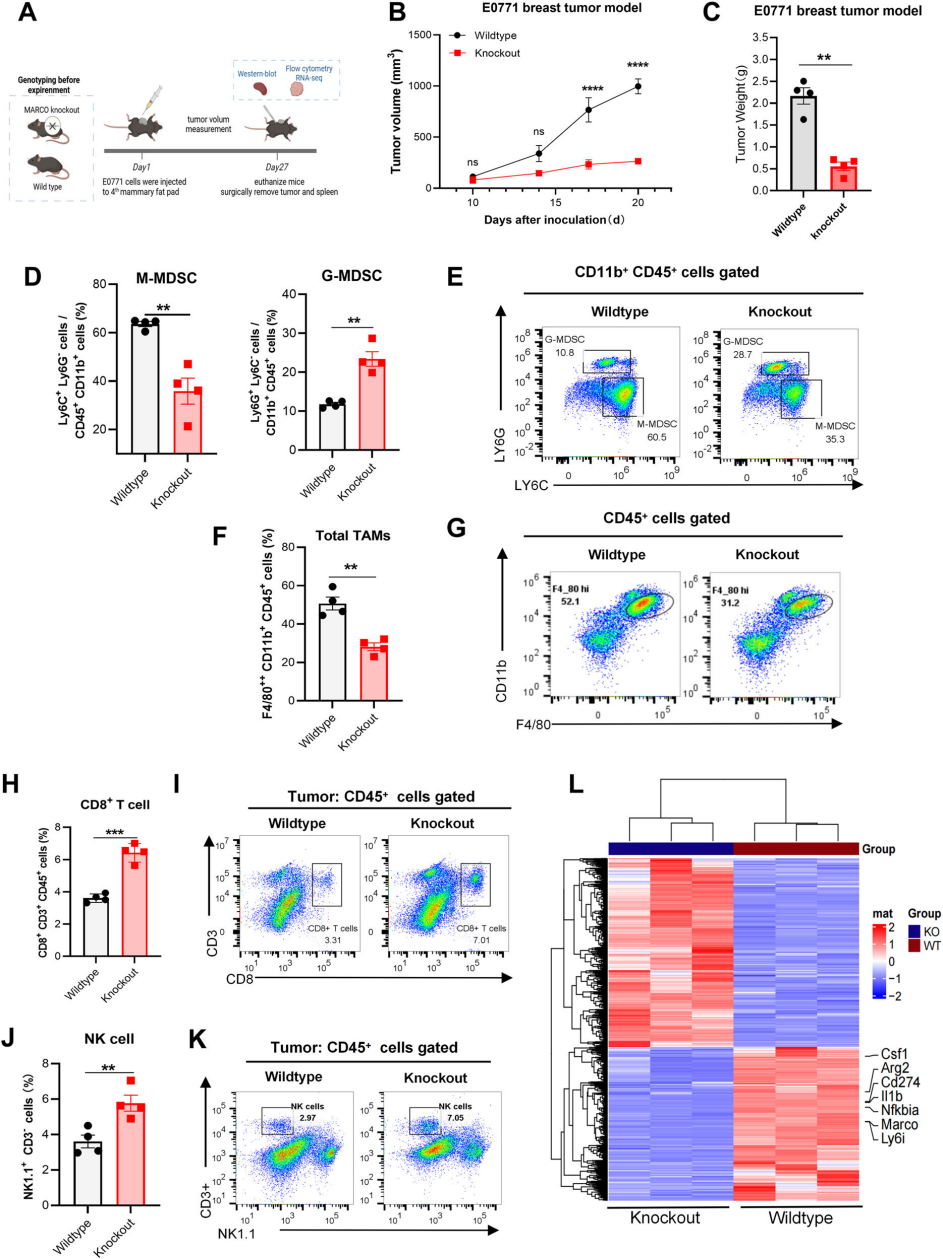
MARCO deficiency prevented tumor growth and altered the characteristics of MDSCs. **A** Schematic of the E0771 mammary tumor model and period of tumor volume measurement. (*n* = 4 for both wild-type and MARCO Knockout groups littermate controls). **B** The growth curve of E0771 breast tumors. Data was shown as mean ± SEM. with a CI of 95%. We analyzed tumor growth differences between the two groups across multiple time points using two-way ANOVA (factors: Group × Time) with Šídák’s multiple comparisons test to show the difference between groups at the same time point. **C** The tumor weight at the experimental endpoint. **D**, **E** Proportions and representative flow cytometry data of G-MDSCs and M-MDSCs in tumor tissue. **F**, **G** Proportions and representative flow cytometry data of TAM. **H**, **I** Quantification and representative flow cytometry data of tumor-infiltrating CD8^+^ T cells. **J**, **K** Quantification and representative flow cytometry data of tumor-infiltrating NK cells. Data in (**C**–**K**) was shown as mean ± SD. with a CI of 95%. Comparisons between the two groups were performed using unpaired two-tailed Student’s t-tests. *p < 0.05, **p < 0.01, ***p < 0.001, ****p < 0.001, ns not significant. **L** MDSCs were isolated from the MARCO deficiency and wild-type group (*n* = 3 for each group) for RNA sequencing analysis. The heatmap showed the differentially expressed genes between knockout MDSCs and wild-type MDSCs.

To investigate the effect of MARCO on tumorigenesis, we constructed MARCO genetic deletion mice. Wild-type mice of the same gender and litter as the control. The genotype of the mice (ranked by ear tags) was determined by western blot (Fig. S3A). Then, E0771 mammary tumor cells were inoculated into the 4^th^ mammary gland of mice. 21 days after inoculation, the mice were euthanized (Fig. 3A). The results showed that MARCO gene knockout prevented tumor growth (Fig. 3B, C & Fig. S3B). The inhibition effect was observed in the B16-F10 melanoma model as well (Fig. S3E–G).

Then, the tumor tissues were collected for flow cytometry analysis. The results indicated that MARCO deficiency reduced the tumor-infiltrated M-MDSCs with the increase of G-MDSCs (Fig. 3D, E). In addition, the proportion of Arg-1^+^ M-MDSC was reduced when MARCO deficiency (Fig. S3C). The total proportion of tumor-infiltrated macrophages (CD45^+^ CD11b^+^ F4/80^+^) was decreased (Fig. 3F, G) in the MARCO-knockout group. Strikingly, the proportion of M2-like TAMs (CD206⁺) was significantly decreased, whereas the proportion of Arg-1^+^ and CD86^+^ TAMs remained no siginicant change (Fig. S3D). A significant increase in the proportion of tumor-infiltrated CD8^+^ T and NK cells was observed in the MARCO deficiency group (Fig. 3H–K).

Further, MDSCs (CD11b^+^ Gr-1^+^) were isolated from tumor tissue for RNA sequencing. The data demonstrated differentially expressed genes (DEGs) between MARCO knockout and wild-type groups (Fig. 3L). MARCO deficiency downregulated *CD274* (PD-L1), *Csf1* (macrophage colony-stimulating factor), *II1b* (IL-1β), and *Nfkbia* (NF-κB inhibitor), which indicates that MARCO deficiency influenced myeloid cell differentiation and disrupted pro-inflammatory pathways. Collectively, these data indicate that MARCO deficiency prevents tumor growth by inhibiting tumor-infiltrated MDSCs and TAMs and increasing CD8^+^ T cells and NK cells. Meanwhile, MARCO deficiency disrupts immunosuppressive pathways and ultimately leads to enhanced anti-cancer immunity.

### MARCO downregulation inducing antibody inhibits the MDSC differentiation

We developed a specific anti-MARCO monoclonal antibody, 2L4-8, which binds to both human and mouse MARCO with high purity and affinity (Fig. S4A, B). We performed a cellular internalization assay and an expression inhibition assay. The results revealed that 2L4-8 induces internalization (Fig. S4C) and subsequent downregulation of MARCO, as evidenced by Western blot and flow cytometry (Fig. S4D–G), consistent with antibody-mediated receptor internalization. Therefore, MARCO downregulation-inducing antibody 2L4-8 not only binds MARCO but actively promotes its removal from the cell surface through an endocytosis-dependent mechanism.

**Fig. 4:**
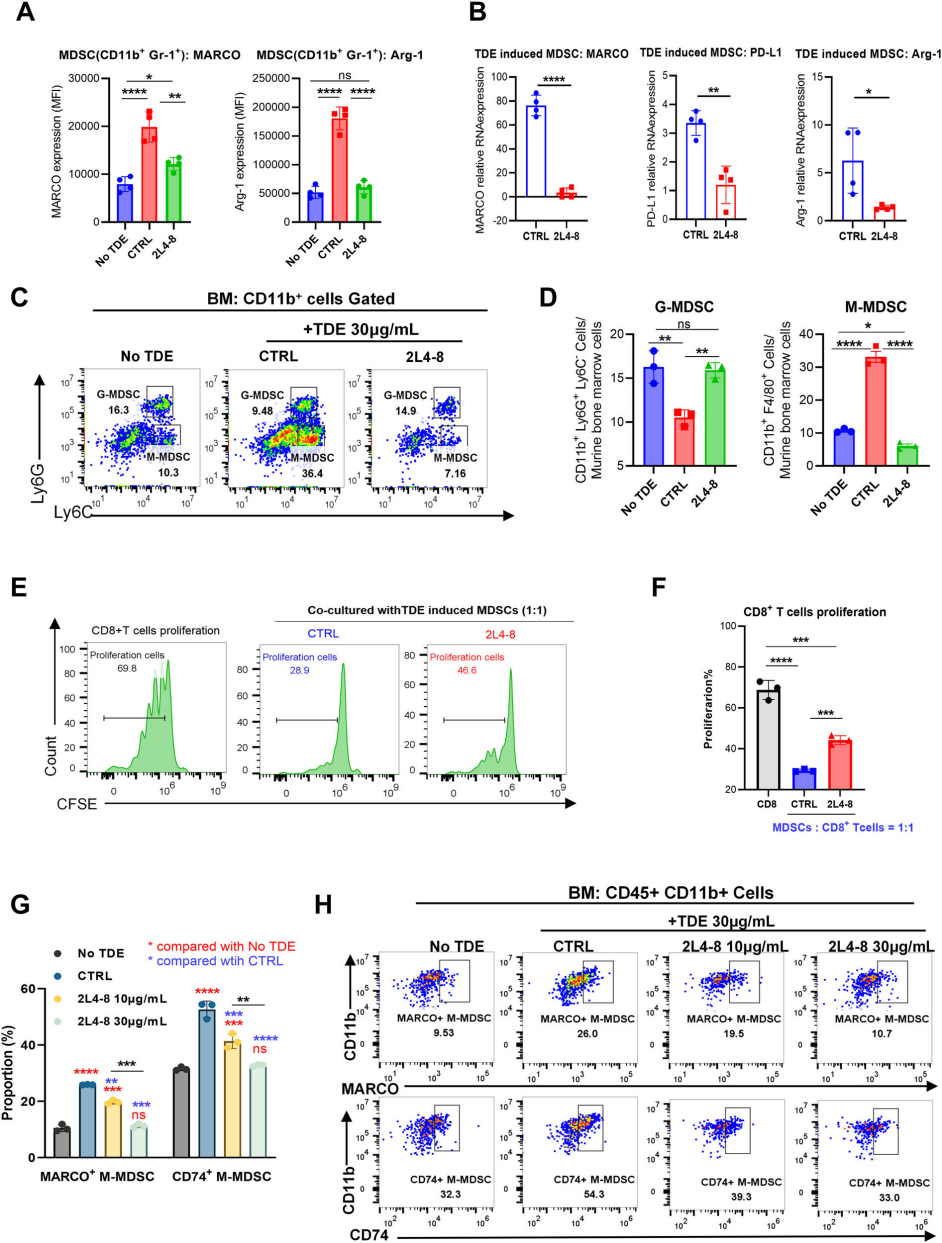
MARCO downregulation-inducing antibody inhibits the MDSC differentiation. Mouse bone marrow cells incubated with E0771 TDEs in the presence of control antibody (CTRL) or MARCO antibody (2L4-8) 30 μg/mL. **A** flow cytometry to detect MARCO and Arg-1 expression on MDSCs. **B** RT-PCR to detect total MARCO, PD-L1, and Arg-1 expression. The experiment contained four biological replicates and was independently repeated at least three times. **C**, **D** Representative flow cytometry data and quantification analysis of the proportion of MDSCs’ sub-populations. **E**, **F** TDE-induced MDSCs were co-cultured with CD8^+^ T cells at a 1:1 ratio with the presence of isotype control and 2L4-8 antibodies for 2 days. Three biological replicates per group. Representative flow cytometry data showed the proliferation of CD8^+^ T cells. Quantification of CD8^+^ T cell proliferation. **G** Quantification data showed the dose-dependent effect of 2L4-8 to inhibit the MARCO and CD74 expression on M-MDSCs. The experiment contained four biological replicates and was independently repeated at least three times. **H** Representative flow cytometry of (**G**). Data were shown as mean ± SD. with a CI of 95%. For experiments with three groups, One-way ANOVA was performed to assess overall differences among the three groups, followed by Tukey’s post hoc test for all pairwise comparisons. For experiments with two groups, an unpaired two-tailed Student’s *t*-test was used. *p < 0.05, **p < 0.01, ***p < 0.001, ****p < 0.0001.

To investigate whether 2L4-8 could counteract TDE-induced MDSC immunosuppression by downregulating MARCO, murine bone marrow cells were treated with E0771 TDEs in the presence or absence of 2L4-8 (30 μg/mL). Flow cytometry revealed that 2L4-8 markedly reduced MARCO expression on MDSCs and concurrently suppressed the production of the immunosuppressive marker Arg-1 (Fig. 4A). Concordantly, qPCR confirmed that 2L4-8 not only downregulated MARCO but also attenuated the expression of Arg-1 and PD-L1 (Fig. 4B). We next investigated whether 2L4-8 alters MDSC differentiation. The result showed that 2L4-8 treatment reduced the M-MDSC proportion induced by E0771 TDEs (Fig. 4C, D) and diminished MARCO and Arg-1 expression in the M-MDSC subgroup (Fig. S4G, H). We also co-cultured CD8^+^ T cells with 2L4-8-treated MDSCs. Notably, 2L4-8 treatment reversed the suppressive capacity of E0771-induced MDSCs, as evidenced by restored CD8⁺T cell proliferation (Fig. 4E, F).

Given that MIF in TDEs implicate MARCO expression on MDSCs, and CD74 serves as the receptor for MIF [25]. We hypothesized that MARCO and CD74 might form a functional axis. Flow cytometry analysis revealed that E0771 TDEs simultaneously upregulated both MARCO and CD74 on MDSCs. Strikingly, 2L4-8 suppressed not only MARCO but also dose-dependently reduced CD74 expression (Fig. 4G, H). In summary, MARCO downregulation, inducing antibody 2L4-8, blocks TDE-induced MDSC differentiation and immunosuppression via inactivation of the MIF-CD74 axis.

### MARCO downregulation antibody attenuates TDE-induced MDSC immunosuppression

**Fig. 5:**
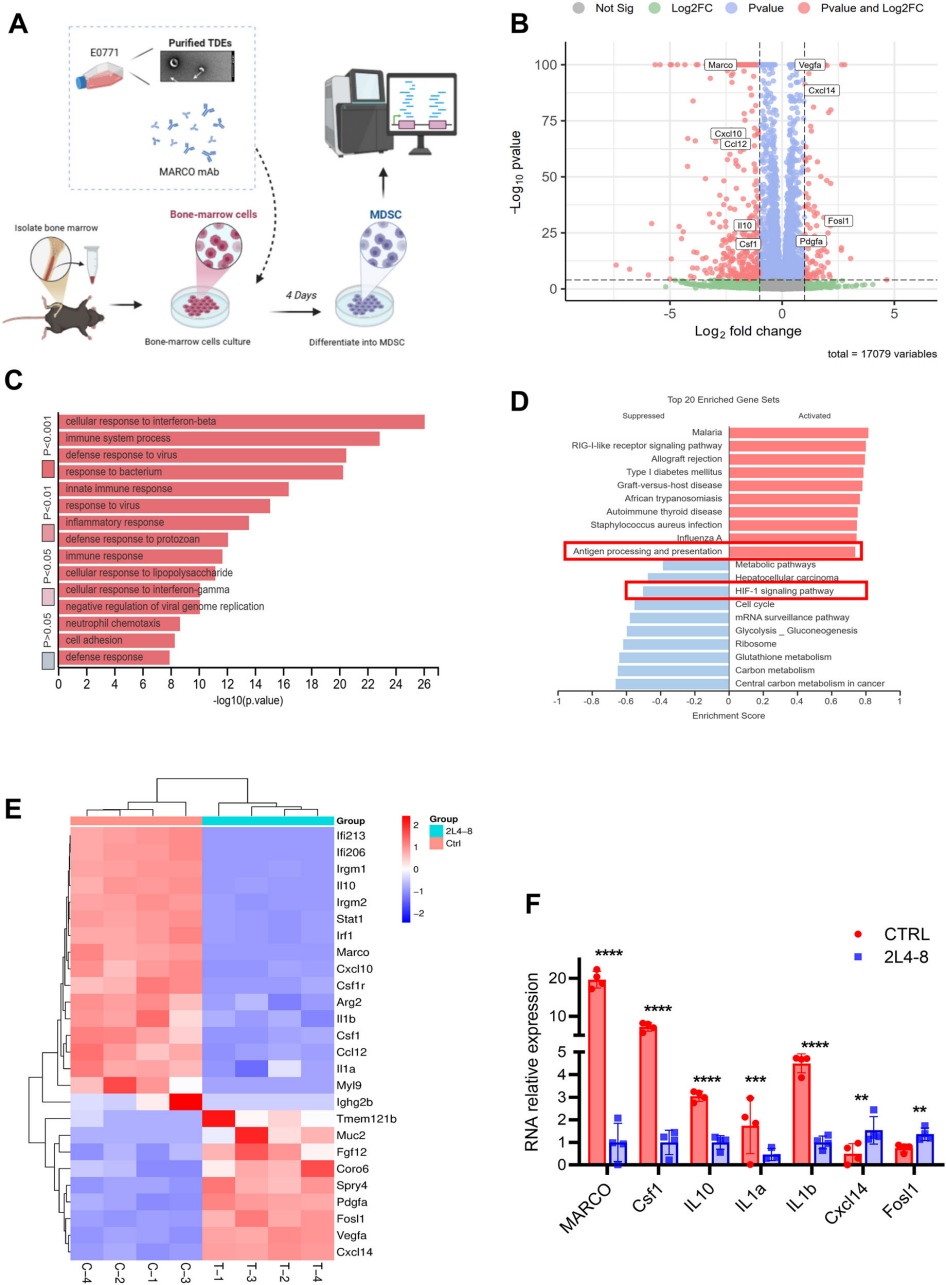
MARCO downregulation antibody attenuates TDE-induced MDSC immunosuppression. **A** Experimental scheme of RNA-seq. Control vs. antibody-treated group (*n* = 4 for each group). **B** Volcano plot displaying differentially expressed genes (DEGs) between treatment and control groups (*n* = 4 each group). The X-axis shows log2 fold change, where positive values indicate higher expression in the treatment group. The Y-axis represents –log10 adjusted *p*-value. Red dots denote significant DEGs (|LFC | > 2, padj < 0.0001). **C** Data from GO enrichment analysis (GO:BP) shows the genes enriched in signaling pathways. **D** GSEA analysis showed the signaling pathway change in the two groups. The results are shown in a bar graph. **E** Heatmap of essential downregulating genes in response to 2L4-8 treatment. **F** All sequenced samples were subjected to RT-PCR to validate the expression of *MARCO, Csf1, IL10, Il1a, Il1b, Cxcl14,* and *Fosl1*. RT-PCR Data was shown as mean ± SD. with a CI of 95%. An unpaired two-tailed Student’s *t*-test was used. **p < 0.01, ***p < 0.001, ****p < 0.0001.

To delineate the molecular mechanisms of TDE-induced MDSC immunosuppression and 2L4-8-mediated MARCO downregulation, E0771 TDE-induced MDSCs were treated with 2L4-8 or control antibody for 4 days. Then, CD11b^+^ Gr-1^+^ MDSCs were isolated for RNA-seq analysis (Fig. 5A).

The volcano plot demonstrated that TDE-induced MDSCs showed a highly immunosuppressive state by upregulating *MARCO, Csf1* (driving immunosuppressive myeloid cell survival)*, IL10* (suppressing cell cytokines), and *Arg-2* (impairing T cell function via arginine depletion). Conversely, TDEs suppress pro-inflammatory responses by the downregulation of *Fosl1* (linked to oncogenic stress), *Pdgfa* (angiogenesis promoter), and *Cxcl14* (chemokine for immune cell recruitment) (Fig. 5B). GO analysis showed 2L4-8 treatment suppressed genes in the cellular response to the IFN-β pathway (Fig. 5C), and GSEA analysis indicated the activation of antigen presentation and the inhibition of HIF-1 signaling after 2L4-8 treatment (Fig. 5D).

Heatmap clustering further highlighted significant transcriptional changes in MDSCs following 2L4-8 treatment, featuring downregulation of immunosuppressive mediators (*Csf1, IL1*0), IFN-β-related genes (*Irgm1, Irgm2, Irf1*), and pro-inflammatory cytokines (*IL1a and IL1b*), while revealing upregulation of immunostimulatory factors *CXCL14* and *Fosl1* (Fig. 5E). These findings were quantitatively validated by RT-qPCR, confirming the decreased expression of *MARCO, Csf1, IL10*, and *IL1a/b* alongside elevated *CXCL14* and *Fosl1* levels (Fig. 5F). These findings unveil that 2L4-8 disrupts MARCO-dependent immunosuppression on MDSCs by modulating immunosuppressive mediators, related with IFN-β and HIF-1 pathways and restoring antigen presentation.

### The MARCO antibody 2L4-8 enhanced the anti-tumor immune response of PD-1 therapy

Previous studies have reported that targeting MARCO antibodies has anti-tumor effects [21, 26, 27]. Here, we demonstrated that 2L4-8, a novel MARCO monoclonal antibody, effectively suppresses tumor growth in a murine E0771 breast cancer model and B16-F10 melanoma model (Fig. S5C–E).

**Fig. 6:**
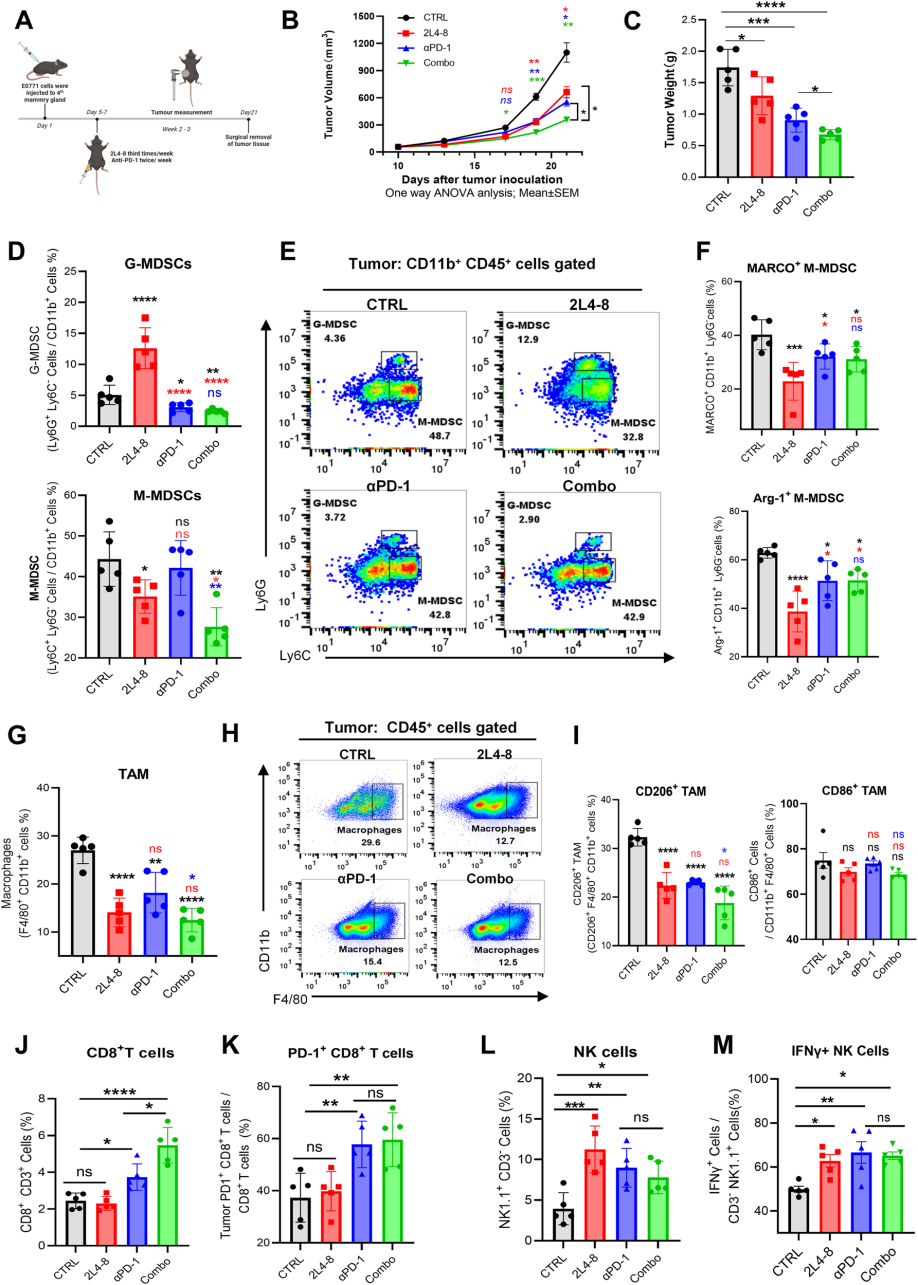
The MARCO antibody 2L4-8 enhanced the anti-tumor immune response of PD-1 therapy. **A** Schematic of the E0771 mammary tumor model and treatment regimen from day 9 to 21 after inoculation. *N* = 5 for each group. **B** E0771 tumor growth in response to different treatments. Data was shown as mean ± SEM with a CI of 95%. The tumor growth differences between the two groups across multiple time points were analyzed by two-way ANOVA (factors: Group × Time) with Šídák’s multiple comparisons test to show the difference between groups at the same time point. **C** Tumor weight of the E0771 tumor. **D** Proportions of tumor-infiltrating G-MDSCs and M-MDSCs in each group. **E** Representative flow cytometry data of MDSC subtypes. **F** Proportion of MARCO^+^ M-MDSC and in Arg-1^+^ M-MDSC each group. **G** Percentage of tumor-infiltrating TAM in each group. **K** Representative flow cytometry data of TAMs. **H** Representative flow cytometry data of TAMs. **I** Percentage of tumor-infiltrating CD206^+^TAM and CD86^+^ TAM in each group. **J**, **K** Percentages of tumor-infiltrating CD8^+^ T cells and PD-1^+^ CD8^+^ T cells in each group. **L**, **M** Percentages of tumor-infiltrating NK cells and IFN-γ^+^ NK cells in each group. The weight of the tumor and flow cytometry data was analyzed using one-way ANOVA with Tukey’s multiple comparisons test for pairwise comparisons between groups. *p < 0.05, **p < 0.01, ***p < 0.001, ****p < 0.001. ns: no significance.

Our findings demonstrate that MARCO inhibition via 2L4-8 or genetic knockout consistently reduced PD-L1 expression on MDSCs, suggesting a combinatorial potential with PD-1 blockade. To evaluate whether 2L4-8 enhances the efficacy of anti-PD-1, E0771 breast tumor-bearing mice (tumor volume ~50 mm³) were randomly divided into four groups: isotype control (CTRL), 2L4-8 monotherapy, anti-PD-1 monotherapy, and anti-PD-1 combined with 2L4-8 (Combo). We observed that 2L4-8 or anti-PD-1 alone exhibited comparable tumor suppression, while the combination group showed significantly enhanced anti-tumor activity compared to monotherapies (Fig. 6B, C), underscoring their synergistic potential.

To further elucidate the immune remodeling in the TME, we performed flow cytometry on tumor-infiltrating immune cells. 2L4-8 monotherapy selectively increased G-MDSCs while reducing M-MDSCs, whereas anti-PD-1 alone had minimal impact on MDSC subsets (Fig. 6D, E). Flow cytometry analysis using Ly6G combined with MARCO or Arg-1 revealed that treatment containing 2L4-8 decreased the frequency of MARCO^+^ M-MDSCs and immunosuppressive Arg-1^+^ M-MDSCs (Fig. 6F & Fig. S5A, B). Further analysis revealed that both 2L4-8 and anti-PD-1 suppressed immunosuppression CD206^+^ TAMs but had no significant effect on CD86^+^ TAMs (Fig. 6G–I). Notably, 2L4-8 alone did not alter CD8^+^ T cell infiltration; however, when combined with anti-PD-1, the proportion of CD8^+^ T cells significantly expanded without change in PD-1 expression compared to anti-PD-1 monotherapy (Fig. 6J, K). Additionally, both 2L4-8 and anti-PD-1 increase the proportion of NK cells and IFNγ^+^ NK cells in the TME (Fig. 6L, M). Collectively, our findings provide strong evidence that MARCO inhibition significantly enhances the anti-tumor immune response of anti-PD-1 immunotherapy by suppressing MARCO expression and MDSC differentiation, altering TAM properties, and promoting the infiltration of CD8^+^ T cells and NK cells.

## Discussion

In the present study, we uncovered a critical role of MARCO in MDSCs. First, we identified that MARCO was expressed on M-MDSC in TME. Then, we demonstrated that MARCO is essential for TDE-induced MDSC differentiation and immunosuppression. We also claimed that MIF in TDE is the main component causing MDSC differentiation and MARCO upregulation. Furthermore, MARCO deficiency prevented tumor growth and was associated with significant changes in MDSCs. To this end, we developed a novel MARCO downregulation antibody 2L4-8 with high affinity and specificity. We observed that 2L4-8 inhibited TDE-induced MDSC differentiation and immunosuppression by downregulating MARCO expression, which was confirmed by RNA-seq analysis. Finally, we validate the anti-tumor effect of 2L4-8 treatment and prove that 2L4-8 significantly enhances the anti-tumor activity of anti-PD-1 therapy.

MARCO, traditionally recognized as a macrophage-specific receptor highly expressed on tumor-associated macrophages (TAMs) in various malignancies, including breast cancer, melanoma, pancreatic cancer, and hepatocellular carcinoma, has recently emerged as a molecule of interest in broader myeloid cell populations. Evidence from genome-wide gene expression profiling has revealed MARCO expression in dendritic cells (DCs) [28–30]. While recent studies have demonstrated that pancreatic ductal adenocarcinoma (PDAC)-conditioned MARCO^+^ macrophages exhibit MDSC-like characteristics [31], the precise expression profile and functional significance of MARCO in MDSCs remain incompletely characterized. In the present study, we provide direct evidence of MARCO expression in MDSCs, particularly the monocytic subset (M-MDSCs) in human breast cancer. It is reported that tumor-secreted extracellular vesicles (EVs) promote MDSC differentiation [32, 33]. Using breast cancer-derived exosomes to induce murine bone marrow or human PBMC, we observed that TDEs not only enhanced MDSC differentiation but also upregulated MARCO expression.

Furthermore, we identified MIF as a critical exosomal component mediating MARCO upregulation on MDSCs. Building on our previous findings that MIF in TDEs promotes MDSC differentiation in TME [24], we demonstrated that MIF knockdown or MIF inhibitor IPG1576 significantly attenuated MDSC differentiation and MARCO upregulation. Furthermore, TDE-induced MDSC exhibited upregulation of CD74, the cognate MIF receptor [34]. Intriguingly, MARCO downregulating antibody 2L4-8 reduced CD74 surface expression. These findings reveal the molecular crosstalk linking MARCO to the MIF-CD74 axis that potentiates TME immunosuppression. While the precise mechanism requires further elucidation, our findings provide therapeutic targeting of this axis as a promising immunotherapy in anti-cancer treatment.

Genetic knockout of MARCO induced a profound remodeling of myeloid cells, characterized by the reduction of M-MDSCs alongside the expansion of G-MDSCs. This subset-specific effect aligns with our discovery that TDE-induced MDSCs are predominantly M-MDSCs, which exhibit high MARCO expression. This suggests that MARCO primarily participates in the differentiation of monocytes into MDSCs. RNA-seq results of MARCO deficiency MDSCs revealed that the reduction of M-MDSCs in MARCO deficiency correlates with downregulated *Csf1* expression, a key regulator of monocyte differentiation. Genetic ablation of MARCO also significantly reduced the Arg-1^+^ M-MDSCs. This indicates MARCO regulates MDSC differentiation via promoting M-MDSC expansion and enhancing their immunosuppressive programming. As MARCO is expressed on multiple myeloid cells, including TAM, we also observed the changes in TAMs. We found MARCO deficiency also reduces the total proportion of TAMs and CD206^+^ TAMs, whereas the frequencies of Arg-1^+^ and CD86^+^ showed no significant change. These results suggest that MARCO regulates the polarization or recruitment of M2-like TAMs without globally affecting macrophage activation or arginine metabolism. Notably, in contrast to prior studies showing MARCO antibody promotes M2-to-M1 repolarization [21], this indicates that the function of MARCO in remodeling macrophages only accounts for a part, and its impact on MDSC is also of crucial importance. MARCO deficiency also enhanced cytotoxic lymphocyte infiltration, with elevated CD8^+^ T cells and NK cells. The downregulation of *IL-1β* and *NF-κB* inhibitor in MARCO deficiency further suggests the disruption of pro-inflammatory signaling pathways that typically sustain tumor-promoting chronic inflammation. Together, MARCO deficiency remodels the immunosuppressive TME by reducing M-MDSCs and M2-like TAMs and enhances cytotoxic lymphocyte infiltration.

Targeting MARCO-expressing TAMs induces their functional reprogramming toward immunostimulatory phenotypes, which in turn enhances the activation and cytotoxic activity of CD8^+^ T cells and NK cells [26, 27]. However, little is known about the effects of MARCO blockade on MDSCs. The novel MARCO downregulation inducing antibody 2L4-8 can inhibit TDE-induced MDSC differentiation and reduce Arg-1 and PD-L1 expression on M-MDSCs. TDE-induced MDSCs exhibited potent immunosuppressive activity by inhibiting CD8^+^ T cell proliferation. Importantly, MARCO downregulating antibody reduces the suppression of TDE-induced MDSC to CD8^+^ T cell proliferation, which may be associated with the antibody-mediated reduction of Arg-1 and PD-L1 expression on MDSCs. RNA-seq analysis of E0771 TDE-induced MDSCs treated with 2L4-8 revealed that 2L4-8 treatment inhibited MARCO expression and further affected immunosuppressive markers such as *Csf1, IL10*, and *Arg-2*, while upregulating pro-inflammatory mediators (*Cxcl14, Fosl1, Pdgfa*). 2L4-8 treatment also suppressed IFN-β response pathways (downregulation of *IL1a, IL1b*) and tumor development-related HIF-1 signaling [35, 36], while enhancing the antigen presentation-related pathway. Collectively, the data confirm that MARCO downregulation antibody prevents TDE-induced MDSC differentiation and immunosuppression. However, RNA sequencing data hint at pathways such as antigen presentation. The key molecules within these pathways lack experimental confirmation.

Immune checkpoint blockade targeting PD-1/PD-L1 has demonstrated significant clinical efficacy in malignancies. However, the majority of solid tumors exhibit limited therapeutic responsiveness, with objective response rates (ORRs) remaining below 20% and over 80% of patients failing to achieve a clinically meaningful benefit. MDSCs in the TME are one of the major reasons for the low response rate [11, 37]. Targeted inhibition of MDSC is a promising strategy to enhance the response rate of anti-PD-1/PD-L1 [38–40]. Our therapeutic investigations establish MARCO-targeted immunotherapy as a potent strategy for remodeling immunosuppressive networks. Notably, the data revealed that TDEs induced MDSC differentiation, accompanied by upregulated MARCO and PD-L1 expression, while MARCO deficiency reduced Arg-1^+^ M-MDSC and reduced CD274 (PD-L1) expression. The inhibition of ARG-1^+^ MDSCs and macrophages synergistically enhanced anti-PD-1 therapy [41], and upregulation of PD-L1 is necessary for PD-1 immune therapy. Building on these findings, we combined a MARCO-inhibitory antibody (2L4-8) with anti-PD-1 therapy. Crucially, the combination of 2L4-8 with anti-PD-1 achieved superior tumor control compared to monotherapies. Mechanistically, 2L4-8 operates independently of PD-1 blockade by directly disrupting M-MDSC differentiation and immunosuppression (MARCO and Arg-1 expressions on M-MDSC were reduced by 2L4-8 treatment) while permitting G-MDSC expansion. 2L4-8 also reduces the total TAM population and suppresses M2-like polarization, which is consistent with observations in the MARCO-knockout mouse model, but contrasting with prior reports suggesting that MARCO-targeting antibodies promote M2-to-M1 repolarization [21]. Collectively, our findings provide strong evidence that MARCO downregulating antibody significantly enhances the anti-tumor immune response of anti-PD-1 immunotherapy by suppressing MDSC differentiation, altering TAM properties, and promoting the infiltration of CD8^+^ T cells and NK cells.

Emerging strategies to enhance PD-1/PD-L1 efficacy by targeting myeloid-derived suppressor cells (MDSCs) have gained significant attention. Below, we contextualize our work with two representative examples. First, Wang et al. performed a CRISPR–Cas9 screen to identify a crucial target CD300ld on PMN-MDSC. They observed that knocking out CD300ld induced the expression of PD-1 on T cells and enhanced the therapeutic effect of PD-1 in the B16-F10 model [38]. The study has established a comprehensive mechanistic network and validated CD300ld’s role using both genetic knockout models and antibody blockade, further corroborating these findings in human cancer samples. While our study identifies MARCO as a regulator of MDSC differentiation and immunosuppression, the mechanistic depth and translational validation remain incomplete. Second, the therapeutic strategy with ATRA, a vitamin A derivative, is to reprogram the differentiation state of MDSCs, converting them to a less immunosuppressive and more mature myeloid cell, such as DCs and macrophages. The prior studies suggest that it may synergize with pembrolizumab [42]. The mechanisms underlying ATRA and our MARCO-targeting strategy exhibit fundamental differences. While ATRA drives the differentiation of MDSCs into less immunosuppressive, mature myeloid cells by reducing reactive oxygen species (ROS), our study demonstrates that the MARCO antibody 2L4-8 specifically suppresses M-MDSC expansion without inducing differentiation. Furthermore, as MARCO expression is predominantly restricted to myeloid cells, our strategy demonstrates superior specificity and safety compared to ATRA. Recently, a phase I clinical trial of MARCO antibody (NCT05560191) targeting patients with advanced or metastatic solid tumors is already underway [26, 31].

In conclusion, we uncovered that TDEs induced MDSC differentiation and immunosuppression via MARCO upregulation, with MIF in TDEs identified as a principal mediator of this process. The development of innovative MARCO antibody 2L4-8 effectively countered TDE-induced MDSC differentiation by diminishing MARCO expression and downstream immunosuppressive pathways while restoring antigen presentation capacity. In vivo, genetic knockout or MARCO downregulation antibody significantly attenuated breast tumor progression through inhibition of MDSC and TAM and revitalization of CD8^+^ T cells and NK cells. Notably, combining 2L4-8 with anti-PD-1 therapy achieved superior tumor control compared to monotherapies, underscoring its potential to overcome resistance to checkpoint blockade by simultaneously targeting myeloid suppression. These findings establish MARCO as a potential therapeutic target and provide compelling evidence for the clinical translation of MARCO antibody-based combination immunotherapy.

## Materials and methods

### Animal and human studies

Female C57BL/6 (RRID: CVCL_C0MU) mice and BALB/c (RRID: MGI: 2161072) were purchased from Shanghai Jihui Laboratory Animal Care Co., Ltd. C57BL/6NCya-Marco^em1^/Cya MARCO knockout mice (Cyagen S-knockout-03129) were purchased from Cyagen. Mice were raised under pathogen-free conditions according to local ethical guidelines. All animal studies were approved by the Institutional Committee for Animal Care and Use, Tongji University School of Medicine, and were performed in accordance with the institutional guidelines.

For human studies, we collected breast cancer tissues from six patients in Shanghai General Hospital before any anti-tumor treatment. All participants gave informed consent prior to sample collection. The biopsy tissues were submitted to the Department of Pathology. This study was approved by the Ethics Committee of Shanghai General Hospital, Shanghai Jiao Tong University School of Medicine.

### Cell culture and plasmids

Human and murine breast cancer cell lines. 4T1 (RRID: CVCL_0125), E0771 (RRID: CVCL_GR23), B16-F10 (RRID: CVCL_0159), MDA-MB-231 (RRID: CVCL_0062), CHOK1 (RRID: CVCL_0214) and HEK293T (RRID: CVCL_0063) were purchased from ATCC in 2022. All the cell lines were authenticated by detecting short tandem repeats in the past year. Mycoplasma testing was performed using PCR detection (Millipore Sigma, #MP0035–1KT) every time before using. The cells were cultured according to ATCC standards in DMEM (Gibco,11965092) or RPMI1640 Medium (Invitrogen, 11875-093) supplemented with 10% FBS (Gibco, 10099–141) and 1% penicillin-streptomycin (Gibco, 15140–122). The cultures were performed at 37 °C with 5% CO_2_, and all cells were used within 10 in vitro passages.

All plasmids carrying the genes of interest used in these experiments were constructed according to General Biol’s standard operating procedure (SOP). In brief, the genes of interest were synthesized by General Biol, following the sh-MIF (MIF shRNA-1: 5′GACAGGGTCTACATCAACTAT-3′). human MARCO CDS sequence (CCDS2124.1), mouse MARCO CDS sequence (CCDS15234.1).

Subsequently, the synthesized genes were inserted into the pLVX-Puro vector between the XhoI and EcoRI restriction enzyme cutting sites, resulting in the generation of sh-MIF-pLVX-Puro plasmids, pLVX-human MARCO-Puro plasmids, and pLVX-mouse MARCO-Puro plasmids. The plasmids containing the genes of interest were used for lentivirus packaging in combination with the psPAX2 (Addgene,12260) and pMD2.G (Addgene, 12259) plasmids at a ratio of 5:3:2, following the standard protocol. Briefly, the three plasmids were mixed with Lipofectamine 2000 transfection reagent (Invitrogen, #11668–027), and the plasmid-lipofectamine mixture was added to HEK293T cells when they reached approximately 50–60% density. Incubate for 10 min at room temperature. Four hours later, the medium was replaced for the transfected HEK293T cells, and lentivirus was collected 48 h post-transfection.

Gene-edited cell lines were established through lentiviral transfection assisted by 8 mg/mL polybrene (Genomeditech, GM-040901B). Stable gene-edited cell lines were selected using puromycin treatment for over 2 weeks.

Using these methods, we generated MDA-MB-231-sh-MIF-pLVX-Puro (sh-MIF), MDA-MB-231-scramble-pLVX-Puro (NC). HEK293T-MARCO-pLVX-Puro (h-MARCO293T) and CHOK1-MARCO-MARCO-pLVX-Puro (m-MARCO CHOK1).The efficiency of gene knockdown or overexpression was confirmed using immunoblotting analysis.

### Flow cytometry analysis

Cell line cells or single cells isolated from the tissues were collected at the end of the experiments. Cells were then incubated with 1 mg/mL mFc Block (BD, AB_394656) to prevent nonspecific antibody binding step at 4 °C for 30 min. Subsequently, the cells were incubated at 4 °C for 30 minutes with various fluorescently labeled antibodies for surface staining (Flow cytometry antibodies were listed in Table S1). PBS + 0.5% BSA (Solai bio A8020) to wash cells at least twice. For intracellular staining, the cells were fixed and permeabilized by BD Cytofix/Cytoperm™ fixation and permeabilization solution (AB_2869010) and then incubated at 4 °C for 30 min with intracellular staining antibodies. The cells were then thoroughly resuspended in 100 μL staining buffer prior to flow cytometric analysis (FACS Calibur, BD Biosciences). Data were analyzed using FlowJo_V10 software.

### TDE extraction and characterization

Tumor cells were cultured in a medium containing 10% exosome-free fetal bovine serum (Gibco A2720-801) for at least 48 h before exosome extraction. The tumor supernatant was collected, and exosomes were extracted using the Exoeasy Maxi Kit (Qiagen, # 76064).

Exosome particles were detected using Nanoparticle Tracking Analysis (NTA). The exosome samples were diluted with 1× PBS for direct use in NTA detection using a particle size particle tracking analyzer (PARTICLE METRIX, model: Zeta VIEW S/N 22-756). The results were analyzed using Zeta View 8.05.14 SP7. Transmission Electron Microscopy (TEM) to observe a single particle directly. 5 μL of exosome sample was taken, excess liquid on one side, drop it onto a copper mesh; incubate at room temperature for 5 min. Then, absorb excess liquid on one side and drop 2% uranyl acetate on the copper mesh, incubate it at room temperature for another 1 min. Until the liquid was totally dry, observe the exosomes on a nanotechnology transmission electron microscope (FEI, model: Tecnai G2 Spirit BioTwin) at 80 Kv. Western blot analysis was performed to detect exosomal markers, such as CD81 and CD9. For antibody information and related steps, please refer to the Western blot method.

### Western blotting

Protein extraction: Tissues and cells were lysed by RIPA Lysis Buffer (Beyotime, P0013B) for 30 min on ice. BCA protein concentration determination kit (Beyotime, P0011) to detect sample concentration. SDS-PAGE protein loading buffer (Beyotime, P0015L) was added to the sample, and the sample was boiled at 100 °C for 5 min.

Western Blotting: load 20 μg for each sample and electrophoresis at 110 V for 1 h in running buffer (Tris-MOPS Buffer 10×, Solarbio, #T1510). Proteins were transferred onto PVDF membranes (Thermo Scientific, 88518) at 80 V for 1 h in a Western Rapid Transfer Buffer (Beyotime, P0572-500ml). The membranes were incubated at 25 mL of QuickBlock™ Blocking Buffer (Beyotime, P0228) for 20 min. The membrane was incubated overnight at 4 °C with the primary antibodies. Then incubated with horseradish peroxidase (HRP) goat anti-rabbit IgG (1:3000; Proteintech, SA00001-2) or HRP goat anti-mouse IgG (1:3000; Proteintech, SA00001-1) antibodies for 1 h. Proteins were visualized using a chemiluminescence imager (Tanon, 4600SF). Primary antibodies used are listed in Table S2.

### Cytokines induced macrophages and MDSCs

Bone marrow cells were isolated from the femurs of C57BL/6 mice. For macrophages, cells were first cultured for 4 days with recombinant murine M-CSF (Pepro Tech, 315-02) in RPMI1640 with 20% FBS to M0 macrophages. Then, adding LPS (MCE, # O55:B5) and IFN-γ (Pepro Tech, 315-05) to polarize toward M1, or adding IL4 (Pepro Tech, 214-14) and IL10 (Pepro Tech, 214-10) to polarize to M2 macrophages for 2 days [43].

For MDSCs, bone marrow cells were cultured in mouse G-M-CSF (Pepro Tech, 315-03) and IL6 (Pepro Tech, 216-16) for 4 days. Macrophages and MDSCs were collected on the final day of culture and analyzed using flow cytometry.

### Tumor condition induced TAMs and MDSCs

To induce murine TAM, M0 macrophages were incubated with a mixture of tumor supernatant and fresh RPMI1640 + 20% FBS in a 1:1 ratio or TDE for 2 days. Mouse IgG Isotype Control (MCE, HY-P80757) was used as a control antibody (CTRL), and blocking MARCO antibodies 2L4-8 were added at the same time and incubated for another 2 days as well.

To induce murine MDSCs, murine bone marrow cells from the femurs of C57BL/6 mice were cultured in RPMI1640 medium supplemented with 20% FBS in the presence of M-CSF (Pepro Tech, # 315-02, 20 ng/mL) and TDEs. CTRL and 2L4-8 antibodies were added the next day and incubated for another 3 days. The proportion of MDSCs was determined by flow cytometry. On day 4 of culture, cells were collected for flow cytometry.

For the induction of human MDSCs, CD14^+^ monocytes were isolated from human PBMC. Then, 2 × 10^5^ monocytes were seeded at proper density in 1640 medium containing 20% FBS with the presence of human GM-CSF (Pepro Tech, # 300-03). Different concentrations of TDEs were then added on the first day. CTRL and 2L4-8 antibodies were added at the same time as TDE addition and incubated for another 3 days. The MDSCs were determined by flow cytometry at day 4.

### Anti-MARCO antibody production and function assay

For anti-MARCO antibody production, newborn BALB/c mice were vaccinated with 293T cells one or two days after birth. After 6–8 weeks, human MARCO overexpressing 293T cells were injected through the abdomen and pedicle every two weeks. Mouse spleen cells were mixed with cultured malignant myeloma cells at a certain proportion. Within 1–2 days of selective cultivation with HAT, a large number of tumor cells die, and after 3–4 days, the tumor cells disappear. Small colonies of round and transparent hybridoma cells resembling myeloma cells were observed. Hybridoma cells can begin screening for antibody activity two weeks after fusion. Culture supernatants were subjected to cell-based ELISA for reactivity with h-MARCO293T cells and m-MARCO CHOK1 cells. The supernatants of the positive clones selected by ELISA were further confirmed by flow cytometry. Limited dilution method for monoclonalization culture of hybridoma cells secreting antibodies. BALB/c mice were intraperitoneally injected with mono-colonized hybridoma cells. About 1–2 weeks later, a large amount of monoclonal MARCO antibodies could be obtained using a syringe to extract ascites. The antibodies were isolated by standard protein purification techniques using G-protein-specific separation columns. Antibody purity (>95%) was confirmed by reduced SDS-PAGE (45/25 kDa)

For the affinity assay of anti-MARCO antibodies, approximately 5 × 10^5^ MARCO overexpressing cells were blocked with TruStain FcXTM (422302, BioLegend) before being incubated with the test antibodies diluted from 225 μg/mL to 0.00381 μg/mL with buffer (PBS + 0.5% BSA) at 4 °C for 30 min. Then, cells were incubated with a secondary antibody (115-545-003, Jackson) for 30 minutes at 4 °C. Flow cytometry analysis to detect MARCO expression at the FITC channel.

For the antibody internalization assay, MARCO overexpressing cells were incubated with detected MARCO antibodies on ice for 1 h at 4 °C and 37 °C, respectively. Cells were then incubated with the secondary antibody Alexa Fluor488 Goat Anti-Mouse (Jackson, #AB_2338840) for 40 min. Flow cytometry was used to detect the fluorescence. The internalization ratio was shown by MFI reduction in the antibody internalization group (incubated at 37 °C) compared to the control group (incubated at 4 °C).

For the MARCO expression inhibition assay, MARCO overexpressing cells were treated with 20 μg/mL candidate MARCO antibodies. After 6 h, 24 h, and 48 h, the cells were collected for flow cytometry and for immunoblotting at only 48 h.

### CD8^+^ T cell proliferation assay

CD8^+^ T cells were isolated from the spleen of C57BL/6 mice by positive selection with CD8 (TIL) microbeads, mouse (Miltenyi # 130-116-478). CD8^+^ T cells were then labeled with the fluorescent Cell Trace CFSE (Invitrogen, C34554) and stimulated by CD3/CD28 beads (Gibco, # 11456D). Macrophages or MDSCs were co-cultured with CD8^+^ T cells for 2 days at a 1:1 ratio in the presence of recombinant murine IL-2 (Peptrotech, # 212-12, 10 ng/mL). The proliferation of CD8^+^ T cells was evaluated by flow cytometry. CSFE-labeled cells were detected by the FITC channel.

### Anti-cancer effect of MARCO mAb 2L4-8 on murine model

#### Model construction

5 × 10^5^ E0771 tumor cells were injected into the 4th mammary fat pad for each 8 weeks C57BL/6 mouse. 5 × 10^5^ 4T1 tumor cells were injected into the 4th mammary fat pad of female mice at the age of 8 weeks. For the B16-F10 melanoma model, 2 × 10^5^ B16-F10 cells for each C57BL/6 mouse were injected subcutaneously in the left axillary region. The tumor-bearing mice were randomly allocated into experimental groups using a computer-generated randomization table when tumors reached ~80 mm³, with equal numbers of animals per group and comparable mean tumor volumes (inter-group difference <1 mm³) at baseline. A minimum of four mice per group were maintained. Mice were excluded when the tumor failed to grow or volumes were outside ±2 SD of the group mean.

#### Measurement and treatment

The body weight and tumor volume were measured every 2 days after grouping (V = 0.5 × a × b^2^). The operator was blinded to group assignments throughout the study. MARCO mAb was injected i.p. every three days. 3 mg/kg anti-mouse PD-1 (CD279; Rat IgG2a) from Selleck (Cat. A2122) was injected i.p. every 6 days.

#### Euthanasia and the collection of tissues

The mice were euthanized when the tumor volume reached approximately 1500–2000 mm^3^. Specimens were systematically arranged by ear tag number in groups. Both operators remained blinded throughout sample processing and data collection. The tumor tissues were collected and stored in cold PBS containing 0.5% BSA. Photographed and recorded.

#### Preparation of single-cell suspension

The tissue was cut into small pieces in cold PBS containing collagenase type 1 (SANGON biotech, a004194-0001) and DNase I Solution (nwbiotch 07900) on ice, and shaken for 30 min at 37 °C. Filtered through a 70 μm screen and lysed red cells using red blood cell lysis buffer (SANGON biotech, b541001-0100). Filtered again and collected single-cell suspensions for flow cytometry.

### RNA sequencing

#### Sample preparation

Tumor-infiltrated MDSCs were isolated from murine E0771 tumor tissues (wild-type *n* = 3 and MARCO-KO *n* = 3) by magnetic-activated cell sorting (MACS) using Miltenyi Myeloid-Derived Suppressor Cell Isolation Kit (130-094-538). Then, cells were quickly frozen in liquid nitrogen in Trizol. TDE-induced MDSCs were collected in TRIzol and quickly frozen.

##### Construction of RNA sequencing libraries and sequencing

Total RNA was extracted from the samples using TRIzol reagent (Invitrogen). The RNA quality was checked with an Agilent 2200 and kept at −80 °C. RNA with an RNA integrity number (RIN) > 7.0, which is acceptable for complementary DNA (cDNA) library construction. cDNA libraries were constructed for each RNA sample using the VAHTS Universal V6 RNA-seq Library Prep Kit for Illumina (Vazyme, Inc.) according to the manufacturer’s instructions. Generally, the protocol consists of the following steps: Poly-A containing mRNA was purified from 1 μg total RNA using oligo (dT) magnetic beads and fragmented into 200–600 bp using divalent cations at 85 °C for 6 min. The cleaved RNA fragments were used for the first- and second-strand complementary DNA (cDNA) synthesis. The dUTP mix was used for second-strand cDNA synthesis, which allowed for the removal of the second strand. The cDNA fragments were end-repaired, A-tailed, and ligated with the indexed adapters. The ligated cDNA products were purified and treated with uracil DNA glycosylase to remove second-strand cDNA. Purified first-strand cDNA was amplified using PCR to create cDNA libraries. The libraries were quality controlled with Agilent 2200 and sequenced by DNBSEQ-T7 on a 150 bp paired-end run.

##### RNA sequencing mapping

Before read mapping, clean reads were obtained from the raw reads by removing the adapter sequences and low-quality reads. Clean reads were aligned to the mouse genome using Star [44]. HTseq [45] was used to get gene counts, and the FPKM method was used to determine the gene expression.

Dif-Gene Analysis: We applied the DESeq2 algorithm [46] to filter the differentially expressed genes, after the significant analysis, *P*-value and FDR analysis [47] were subjected to the following criteria (Fold Change >2 or <0.5, *P*-value < 0.05, FDR < 0.05).

##### Dif-gene analysis

We applied the DESeq2 algorithm [46] to filter the differentially expressed genes, after the significant analysis, P-value and FDR analysis [47] were subjected to the following criteria (Fold Change >2 or <0.5, *P*-value < 0.05, FDR < 0.05).

##### Go analysis

Gene ontology (GO) analysis [48] was performed to facilitate elucidating the biological implications of the differentially expressed genes in the experiment. GO annotations were downloaded from NCBI (http://www.ncbi.nlm.nih.gov/), UniProt (http://www.uniprot.org/), and Gene Ontology (http://www.geneontology.org/). Fisher’s exact test was used to identify significant GO categories (*p* < 0.05).

##### KEGG pathway analysis

KEGG analysis includes metabolism, membrane transport, signal transduction, and cell cycle pathways [49]. Pathway analysis was used to determine the significant pathways of the differentially expressed genes according to the KEGG database. Fisher’s exact test was used to select the significant pathway, and the threshold of significance was defined as a *P*-value < 0.05.

GSEA: GSEA analyses [50] were performed using fpkm values to identify the most significant pathways following the KEGG gene sets.

### Quantitative RT-PCR

Total RNA was extracted from tissues or cells using the SteadyPure fast RNA extraction kit (Accurate Biology AG21023). Reverse transcribe RNA into cDNA using the Evo M-MLV reverse transcription premix kit. cDNA was amplified and quantified using QuantStudio^TM^ 6 Flex Real-Time PCR System (Applied Biosystems, Shanghai, China) and Taq Pro Universal SYBR qPCR Master Mix (Vazyme, Q712-02).

Glyceraldehyde-3-phosphate dehydrogenase (GAPDH) was used as the endogenous control. Relative fold changes in specific mRNA expression were calculated using the comparative threshold cycle (2^−ΔΔCt^) method. Primers are listed in Table S4.

### Multi-target immunofluorescence (m-IF)

The obtained tumor tissue of breast cancer patients was fixed in 4% paraformaldehyde and made into 4-μm-thick paraffin pathological sections for subsequent multiple immunofluorescence staining. These sections were dewaxed and rehydrated using a gradient of xylene (Sinopharm Chemical Reagent and alcohol). Tissue sections were placed in a box filled with pH 6.0 citric acid repair buffer (Record bio #RC015) and subjected to antigen repair in a microwave at a medium fire for 8 min. After natural cooling, use a solution containing 5% BSA for the blocking step. Then, the slices were incubated with primary antibodies (Table. S3) specific to the target protein at 4 °C overnight. After incubation, the slices were washed three times with PBS for 5 min each. A four-color multiple immunofluorescence assay kit (Record Bio, #RC0086-34RM) was used for labeling all targets in fluorescence. Finally, immunofluorescence images were captured by immunofluorescence microscopy (OLYMPUS, CX23); Case-viewer 2.0 was applied for image processing.

### Statistical analysis

All statistical analyses and graphical representations were performed using GraphPad Prism version 9.0 software. The default data are presented as the Mean ± SD with 95% confidence intervals (95% CI). Non-paired Student’s *t*-test is used for statistical analysis between two sets of data. One-way ANOVA was performed to assess overall differences among more than two groups, followed by Tukey’s post hoc test for all pairwise comparisons. The two-way ANOVA test is used for tumor volume growth curves that are influenced by both time and volume factors. Data in the curve are presented as Mean ± SEM with 95% CI. Sidak’s multiple comparisons test for individual time point comparisons between groups. Dose-response curves were generated via four-parameter logistic (4PL) nonlinear regression. EC_50_ values were derived from the fitted curves with 95% confidence intervals. All experiments were performed with at least three biological replicates or repeated independently on multiple occasions.

## Supplementary information

Supplementary tables S1–S4

Supplementary Figure 1

Supplementary Figure 2

Supplementary Figure 3

Supplementary Figure 4

Supplementary Figure 5

Supplementary Figure Legends

Original blot

## Data Availability

All data generated or analyzed during this study are included in this published article and its supplementary information files.
