# Supplementary material for: MARCO expression on myeloid-derived suppressor cells is essential for their differentiation and immunosuppression
Source: Cell Death Discov. 2025 Jul 22;11:337. doi: 10.1038/s41420-025-02627-1 (PMC12283920; doi:10.1038/s41420-025-02627-1)
Supplement: Supplementary file 1 — Supplementary tables S1–S4 [file 41420_2025_2627_MOESM1_ESM.docx]

**Supplementary tables**

**Supplementary Table.1 Antibodies used in flow cytometry.**

| Antibody | Vendor | Catalog |
| --- | --- | --- |
| FITC anti-mouse CD45 | BD Pharmingen | 553079 |
| PerCP-Cy5.5 anti-CD11b | BD Pharmingen | 550993 |
| PE Anti-mouse F4/80 | Biolegend | 111604 |
| BV421 anti-mouse CD206 | Biolegend | 141717 |
| PE-Cy7 Anti human/mouse Arg1 | Thermo | 25-3697-82 |
| BV510 anti-mouse CD86 | Biolegend | 105040 |
| APC anti-mouse MARCO | R&D systems | FAB2956A |
| BV510 Anti-mouse CD11b | BD Pharmingen | 562950 |
| BV421 anti-mouse Ly6G | BD Pharmingen | 562737 |
| PE anti-mouse Ly6C | BD Pharmingen | 560592 |
| PerCP-Cy5.5 anti-mouse CD74 | BD Pharmingen | 2136177 |
| PE-Cy7 anti-mouse CD3 | Biolegend | 100200 |
| BB700 anti-mouse CD8 | BD Pharmingen | 566409 |
| PE anti-mouse NK1.1 | Biolegend | 156504 |
| APC anti-mouse IFN-γ | Biolegend | 505810 |
| PE anti-human CD11b | BD Pharmingen | 555388 |
| PE-Cy7 anti-human CD33 | Biolegend | 983908 |
| PerCP anti-human CD15 | Biolegend | 323018 |
| APC-Cy7 anti-human CD14 | BD Pharmingen | 557831 |
| APC anti-human MARCO | Invitrogen | 17-5447-42 |

**Supplementary Table.2 Antibodies used in western-blotting.**

| Antibody | Vendor | Catalog | Dilution |
| --- | --- | --- | --- |
| Anti-mouse MARCO antibody | Abcam | ab239369 | 1:1000 |
| Anti-human MARCO antibody F-3 | Santa cruz | sc-398053 | 1:1000 |
| Anti-mouse/human CD81 antibody | Santa cruz | sc-166029 | 1:500 |
| Anti-human/mouse CD9 antibody | Protein Tech | 20597-1-AP | 1:1000 |
| Beta Actin Poly-clonal antibody | Protein Tech | 20536-1-AP | 1:5000 |

**Supplementary Table.3 Antibodies used in IF**

| Antibody | Vendor | Catalog | Dilution |
| --- | --- | --- | --- |
| Anti-human CD68 antibody | Invitrogen | MA5-13324 | 1:1000 |
| Anti-human CD11b antibody | Santa cruz | sc-398053 | 1:1000 |
| Anti-human CD14 antibody | Invitrogen | 16-0149-82 | 1:1000 |
| Anti-human CD15 antibody | Invitrogen | 14-0159-82 | 1:1000 |
| Anti-human MARCO antibody | Sigma | HPA063793 | 1:1000 |

**Supplementary Table.4 Mouse primer sequences for quantitative real-time PCR**

| Gene | Forward primer sequence (5’-3’) | Reverse primer sequence (5’-3’) |
| --- | --- | --- |
| GAPDH | AGGTCGGTGTGAACGGATTTG | TGTAGACCATGTAGTTGAGGTCA |
| MARCO | ACAGAGCCGATTTTGACCAAG | CAGCAGTGCAGTACCTGCC |
| IL1b | GCAACTGTTCCTGAACTCAACT | ATCTTTTGGGGTCCGTCAACT |
| IL1a | CGAAGACTACAGTTCTGCCATT | GACGTTTCAGAGGTTCTCAGAG |
| Csf1 | ATGAGCAGGAGTATTGCCAAGG | TCCATTCCCAATCATGTGGCTA |
| MARCO KO 1 | TCTGAATTCCTTTTGGCCTATGTC | TTTTACGGCCCAGCTAGTTCTATT |
| MARCO KO 2 | TCTGAATTCCTTTTGGCCTATGTC | AAATCTCCAGAGCCAGATAAAGGC |
| CXCL14 | GAAGATGGTTATCGTCACCACC | CGTTCCAGGCATTGTACCACT |
| IL10 | GCCCTTCCTATGTGTGGTTTG | TTGAGTTTCCGTACTGTTTGAGG |
| Fosl1 | ATGTACCGAGACTACGGGGAA | CTGCTGCTGTCGATGCTTG |
