# Supplementary figures and images for: MARCO expression on myeloid-derived suppressor cells is essential for their differentiation and immunosuppression
Source: Cell Death Discov. 2025 Jul 22;11:337. doi: 10.1038/s41420-025-02627-1 (PMC12283920; doi:10.1038/s41420-025-02627-1)

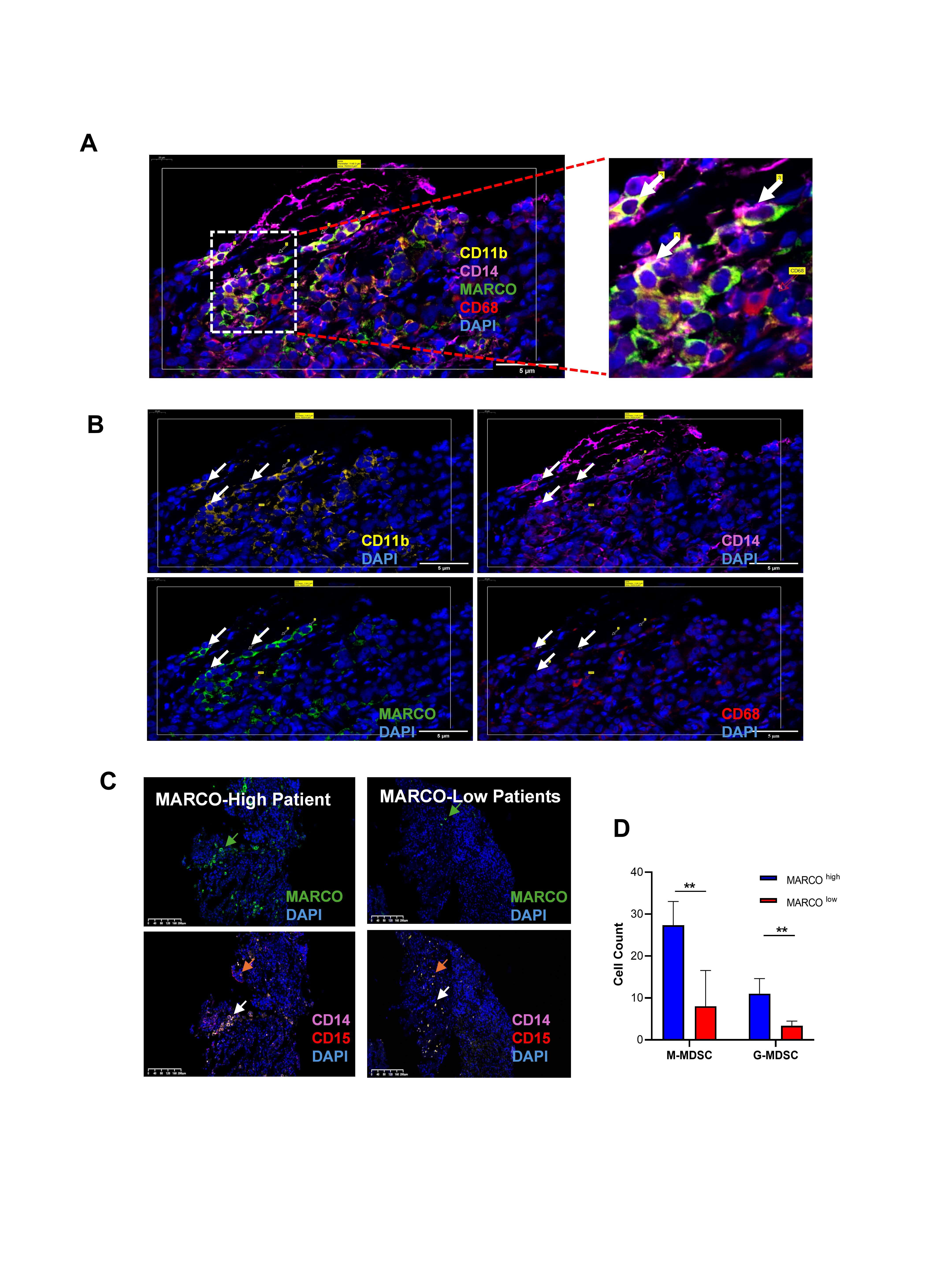

Supplement: Supplementary file 2 — Supplementary Figure 1 [file 41420_2025_2627_MOESM2_ESM.png]

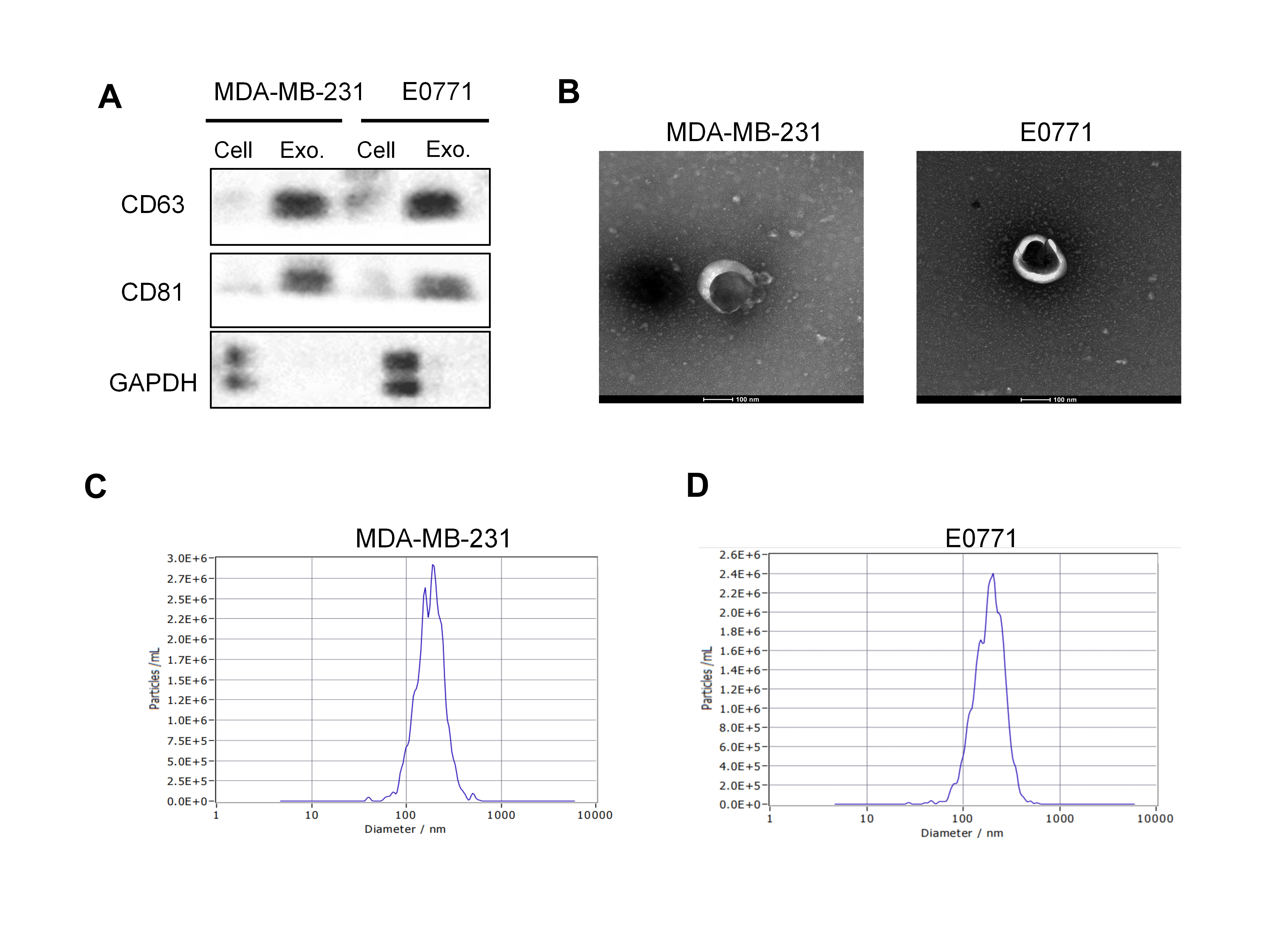

Supplement: Supplementary file 3 — Supplementary Figure 2 [file 41420_2025_2627_MOESM3_ESM.png]

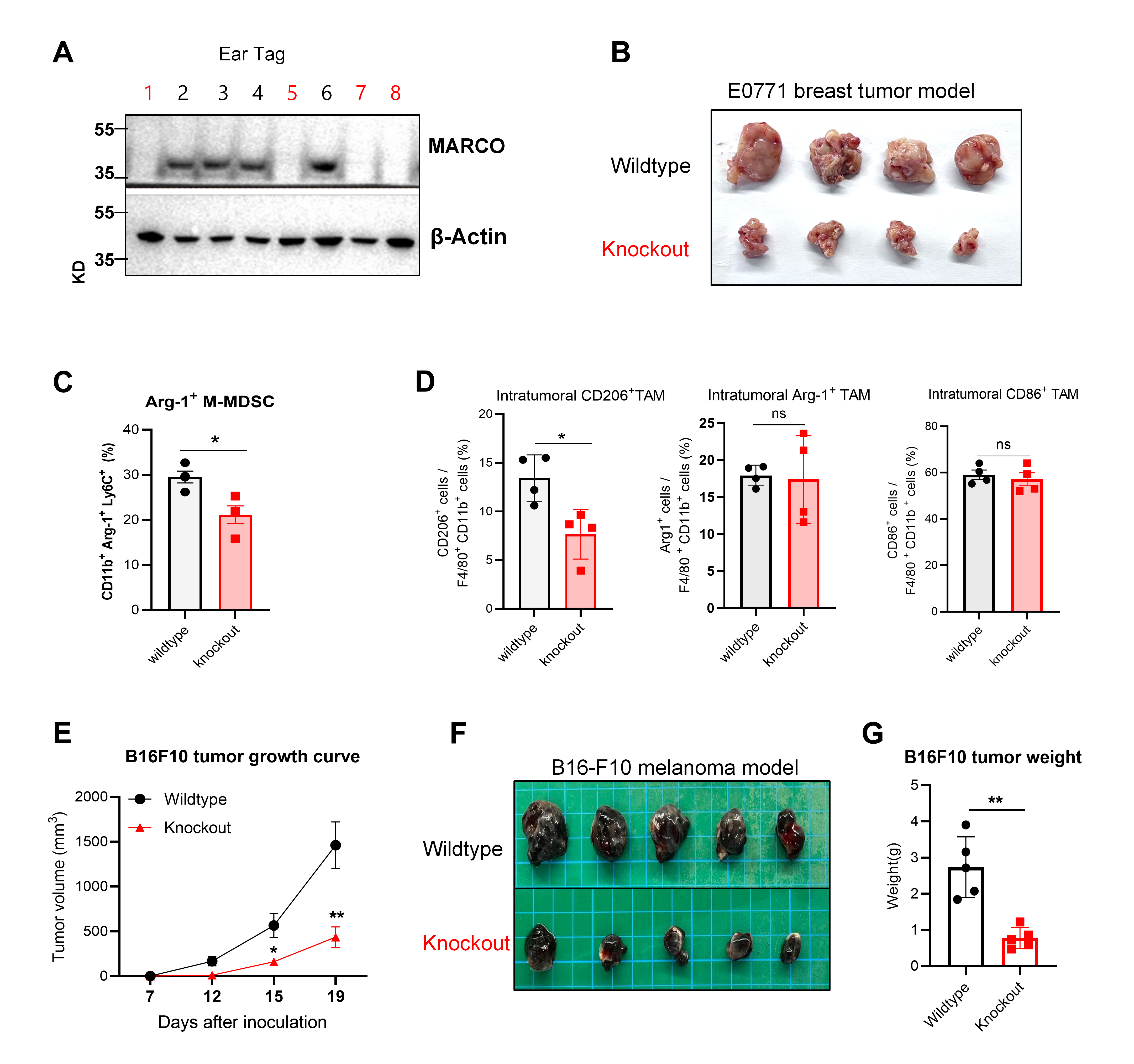

Supplement: Supplementary file 4 — Supplementary Figure 3 [file 41420_2025_2627_MOESM4_ESM.png]

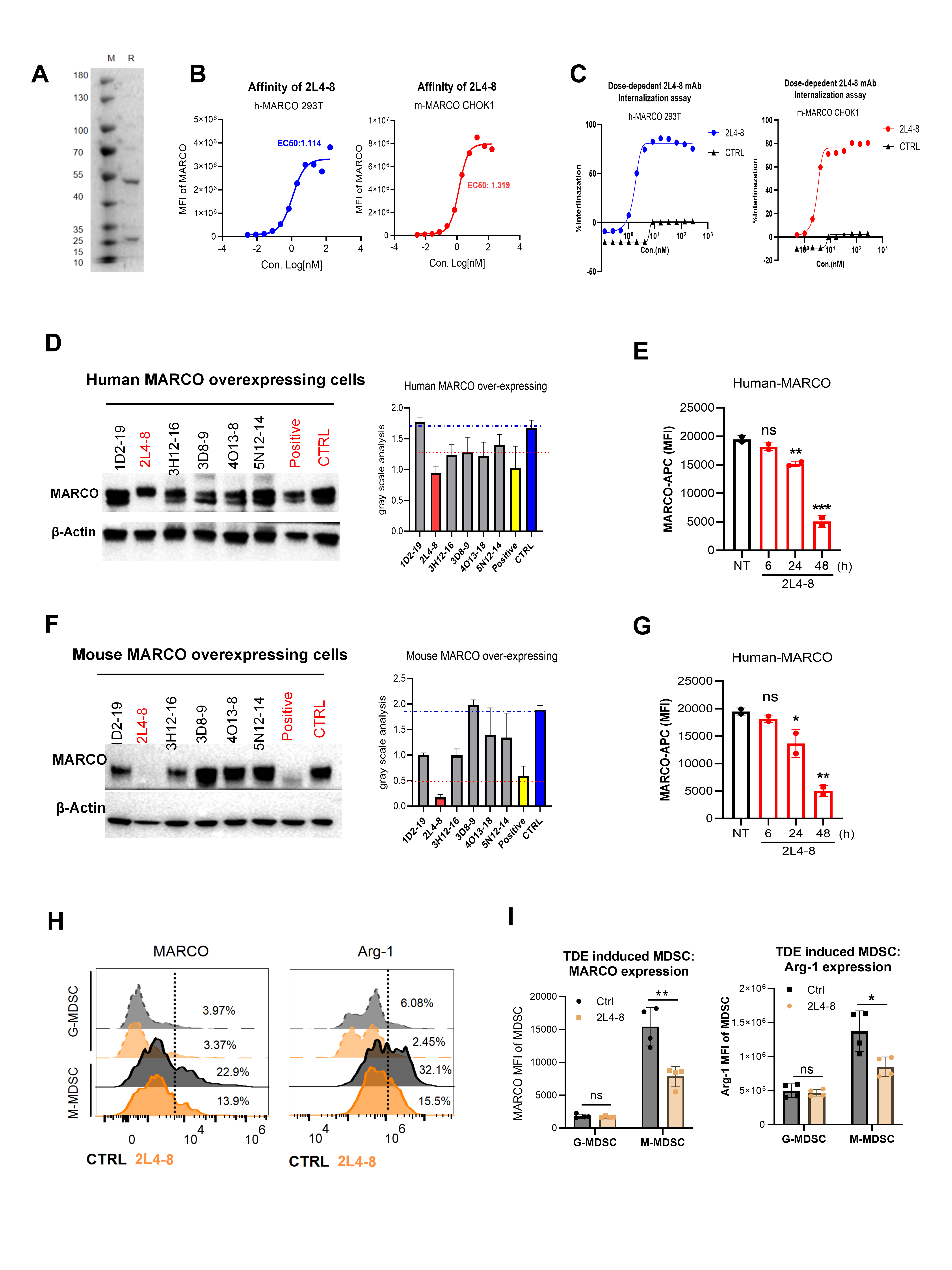

Supplement: Supplementary file 5 — Supplementary Figure 4 [file 41420_2025_2627_MOESM5_ESM.png]

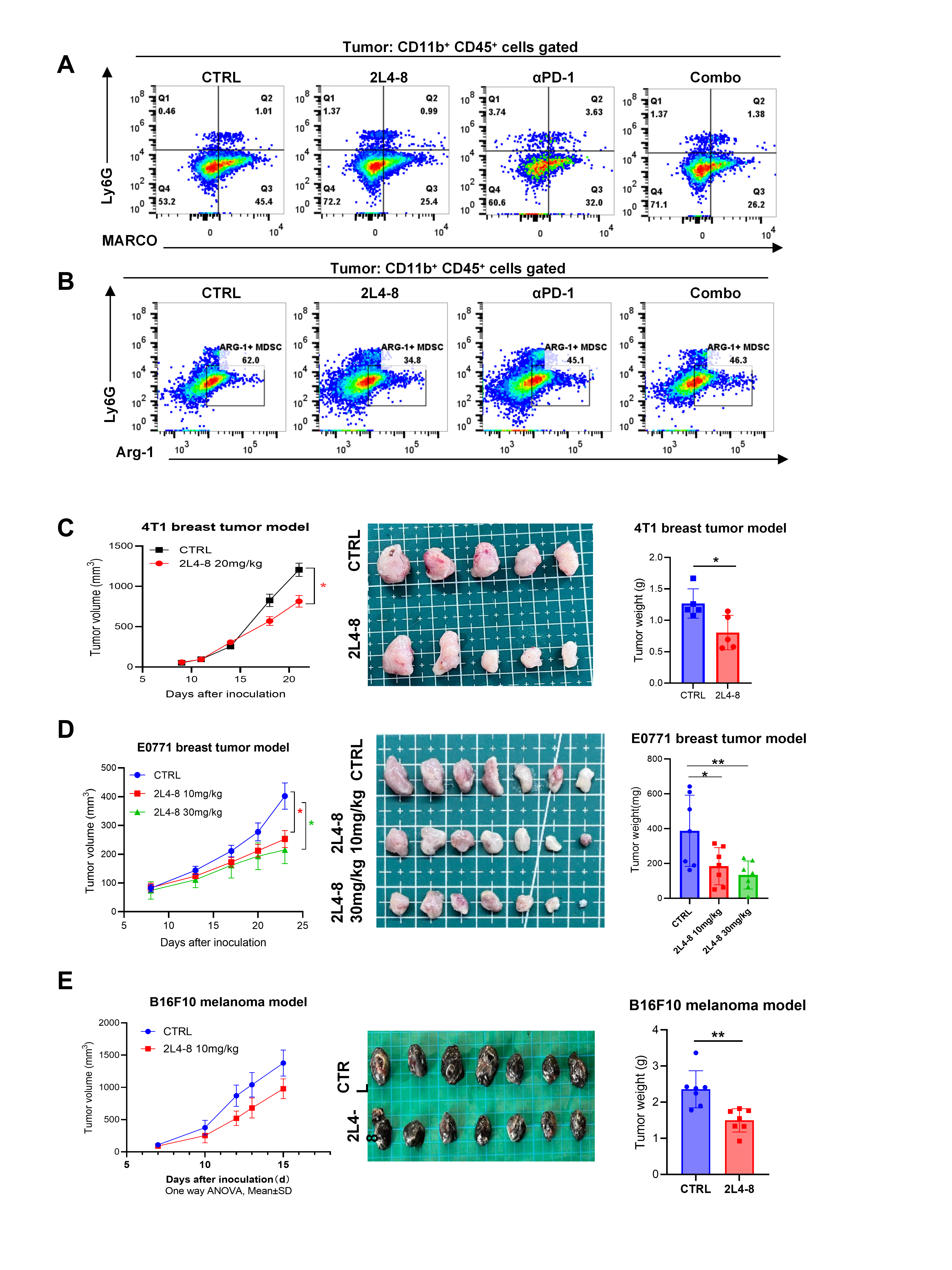

Supplement: Supplementary file 6 — Supplementary Figure 5 [file 41420_2025_2627_MOESM6_ESM.png]

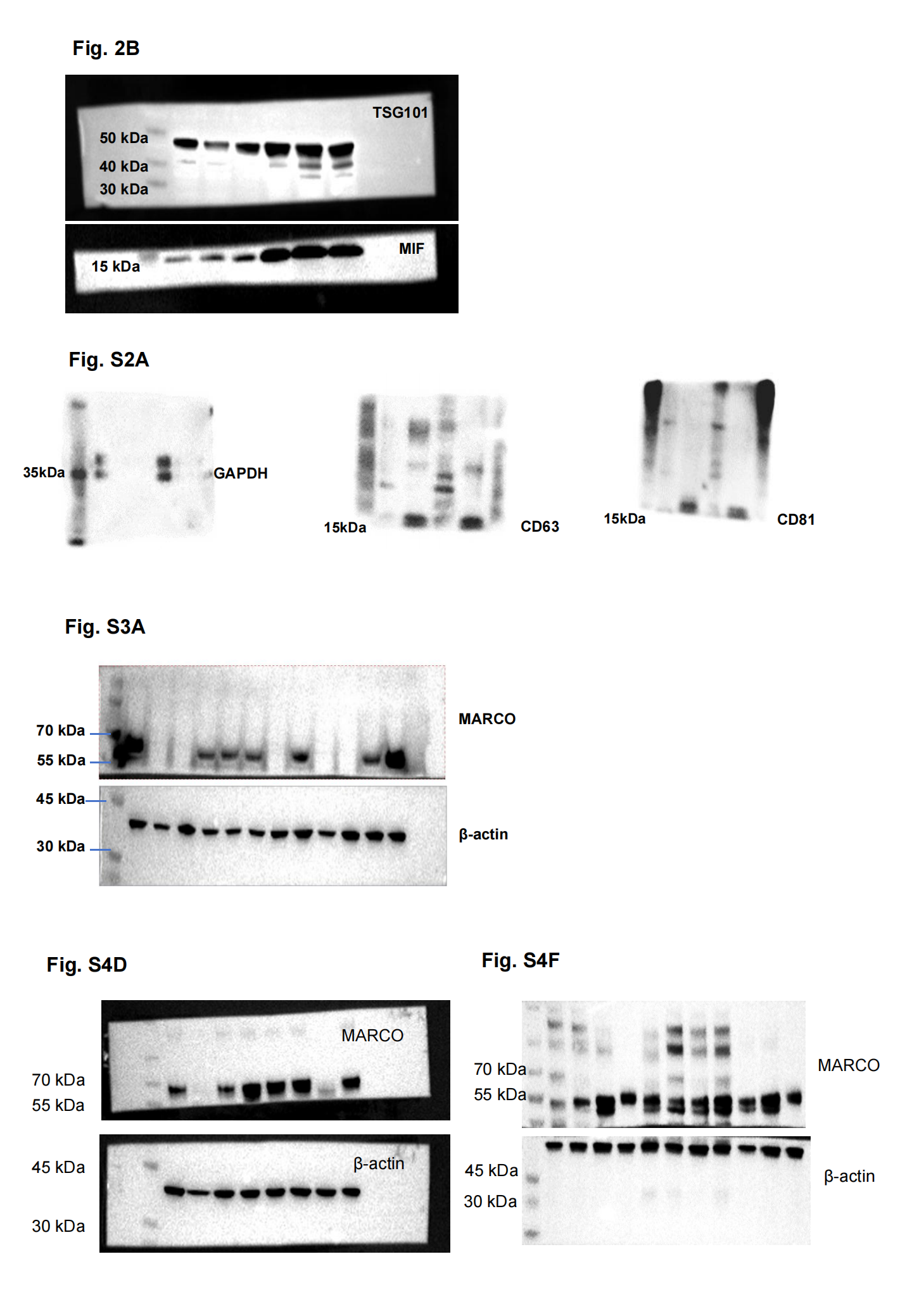

Supplement: Supplementary file 8 — Original blot [file 41420_2025_2627_MOESM8_ESM.png]
