# Supplementary material for: MARCO expression on myeloid-derived suppressor cells is essential for their differentiation and immunosuppression
Source: Cell Death Discov. 2025 Jul 22;11:337. doi: 10.1038/s41420-025-02627-1 (PMC12283920; doi:10.1038/s41420-025-02627-1)
Supplement: Supplementary file 7 — Supplementary Figure Legends [file 41420_2025_2627_MOESM7_ESM.docx]

**Supplementary Figure Legends**

**Fig. S1.MARCO expressed on MDSCs in human breast cancer**

(**A-B)** Multicolor immunofluorescence analysis of a representative human breast cancer tissue section demonstrating co-localization of MARCO with MDSCs. The merged image (left panel) and magnified view (boxed region) reveal spatial overlap between MARCO (green) and MDSC markers: CD11b^+^ (pan-myeloid marker, yellow), CD14^+^ (monocytic lineage, magenta), and CD68^+^ (macrophage marker, red), with DAPI (nuclei, blue). White arrows highlight MARCO^+^ cells within CD11b^+^ CD14^+^ CD68^-^ M-MDSC clusters. **(B)** Individual channel images corresponding to the magnified region in (A):CD11b (yellow), CD14 (magenta), MARCO (green), CD68 (red) and DAPI (blue) are shown. **(C-D)** Multicolor immunofluorescence analysis of total 6 patients demonstrated MARCO^+^ cell infiltration with MDSC infiltration. Based on the median count of MARCO^+^ cells, patients were stratified into MARCO-high (n=3) and MARCO-low (n=3) groups for comparative analysis. **(C)** Representative IF staining in MARCO high expression patient (left) or low expression patient (right) reveals enriched infiltration of M-MDSC: CD11b^+^ CD14^+^ CD15^-^ cells (white arrows) and G-MDSC: CD11b^+^ CD15^+^ CD14^-^ cells (orange arrows). **(D)** Statistical analysis of MDSC subgroup in two group. Data was shown as mean ± SD. with a CI of 95%. Comparisons between the two groups were performed using unpaired two-tailed Student's t-tests. **p < 0.01.

**Fig. S2. Breast cancer TDE identification.**

**(A)** Representative Western blots demonstrate the expression exosomal markers of TDEs isolated from MDA-MB-231 and E0771 breast cancer cells. **(B)** Observation of MDA-MB-231 and E0771 TDEs under the transmission electron microscopy. **(C-D)** NTA detection results of MDA-MB-231 and E0771 TDEs.

**Fig. S3. MARCO knockout identification and related tumor model**

**(A)** Western-blot to detect the expression of MARCO in the spleen of mice (N=4 each group). The sample was ranked by number of ear tag. No.1, 5, 7 and 8 were double identified as MARCO knockout mice. **(B)** Tumor size of E0771 tumors on MARCO knockout and wildtype mice. **(C)** Quantification of Arg-1^+^ M-MDSCs in E0771 tumor tissues in MARCO knockouot and wildtype group. **(D)** Quantification of macrophage Polarization states in E0771 tumor tissues in each group. **(E)** Tumor growth curve, size and weight of B16-F10 model in wildtype and MARCO knockout group. Tumor growth curve was shown as mean ± SEM with a CI of 95%. The differences between the two groups across multiple time points were analyzed by two-way ANOVA (factors: Group × Time) with Šídák's multiple comparisons test. All bar graph was shown as mean ± SD. with a CI of 95%. Comparisons between the two groups were performed using unpaired two-tailed Student's t-tests. *p < 0.05, **p < 0.01, ns: no significance.

**Fig. S4. In vitro profile of MARCO down-regulating inducing antibody 2L4-8.**

(A) Antibody purity was verified by SDS-PAGE under reducing conditions. **(B)** The binding affinity of anti-MARCO antibody 2L4-8 was assessed by flow cytometry on human MARCO over-expressing 293T cells and mouse MACO over-expressing CHOK1 cells. Serial dilutions of 2L4-8 (0.1-100 μg/ml) were tested in single technical replicates at each concentration to establish the binding curve. Following logistic curve fitting, the half-maximal effective concentration (EC50) was calculated. **(C)** The dose-dependent internalization of 2L4-8 antibodies on human MARCO over-expressing 293T cells and mouse MACO over-expressing CHOK1 cells. The curve was fitted by logistic regression model. **(D)** Representative western- blot result to demonstrated the inhibition effect of different clone of anti-MARCO antibody selected by high-affinity on human MARCO over-expressing 293T cells. And relative gray value analysis of each MARCO antibody. **(E)** Flow cytometry result showed MARCO reducing expression at different time point compared to no treated group, 2 replicates at each time point. **(F-G)** The MARCO expression changes in mouse MACO over-expressing CHOK1 cells. **(H)** Representative flow cytometry data demonstrating the proportion of Arg-1 and MARCO expression on MDSCs’ sub-populations after 2L4-8 treatment. **(I)** Quantification data of G.

Data was shown as mean ± SD. with a CI of 95% and individual data points overlaid. One-way ANOVA was performed to assess overall differences among three groups, followed by Tukey's post hoc test for all pairwise comparisons. For experiments with two groups, unpaired two-tailed Student’s t-test was used. ******p* < 0.05, *******p* < 0.01, ********p* < 0.001, *********p* < 0.0001, ns: no significance.

**Figure. 5 Anti-tumor effect of 2L4-8 on murine tumor model.**

**(A-B)** Supplement to the quantification resulted showed in Fig. 6F. **(A)** Representative flow cytometry data demonstrated the proportion of MARCO^+^ M-MDSC (Q3: MARCO^+^ Ly6G^-^ ) in each group gated by CD11b^+^ CD45^+^ live cells. **(B)** Representative flow cytometry data demonstrated the proportion of Arg-1^+^ M-MDSC in each group gated by CD11b^+^ CD45^+^ live cells. **(C)** Dose-dependent tumor inhibition effect of 2L4-8 antibody on E0771 breast tumor model with different dose (N=7 for each). **(D)** Tumor inhibition effect of 2L4-8 antibody on 4T1 breast tumor model. **(G)** Tumor inhibition effect of 2L4-8 antibody on B16-F10 melanoma model (N=7 for each).

For bar graph, data was shown as mean ± SD. with a CI of 95% and individual data points overlaid. One-way ANOVA was performed to assess overall differences among three groups, followed by Tukey's post hoc test for all pairwise comparisons. For experiments with two groups, unpaired two-tailed Student’s t-test was used. ******p* < 0.05, *******p* < 0.01. For tumor growth curve, the differences between the two groups were analyzed by two-way ANOVA (factors: Group × Time) with Šídák's multiple comparisons test.
